# Vaccination against swine influenza in pigs causes different drift evolutionary patterns upon swine influenza virus experimental infection and reduces the likelihood of genomic reassortments

**DOI:** 10.3389/fcimb.2023.1111143

**Published:** 2023-03-13

**Authors:** Álvaro López-Valiñas, Marta Valle, Miaomiao Wang, Ayub Darji, Guillermo Cantero, Chiara Chiapponi, Joaquim Segalés, Llilianne Ganges, José I. Núñez

**Affiliations:** ^1^ IRTA, Programa de Sanitat Animal, Centre de Recerca en Sanitat Animal (CReSA), Campus de la Universitat Autònoma de Barcelona (UAB), Barcelona, Spain; ^2^ Unitat mixta d’Investigació IRTA-UAB en Sanitat Animal, Centre de Recerca en Sanitat Animal (CReSA), Campus de la Universitat Autònoma de Barcelona (UAB), Barcelona, Spain; ^3^ WOAH Collaborating Centre for the Research and Control of Emerging and Re-Emerging Swine Diseases in Europe (IRTA-CReSA), Barcelona, Spain; ^4^ WOAH Reference Laboratory for Swine Influenza, Istituto Zooprofilattico Sperimentale della Lombardia ed Emilia-Romagna, Brescia, Italy; ^5^ Departament de Sanitat i Anatomia Animals, Universitat Autònoma de Barcelona, Barcelona, Spain; ^6^ WOAH Reference Laboratory for Classical Swine Fever, IRTA-CReSA, Barcelona, Spain

**Keywords:** swine influenza virus, vaccination, coinfection, virus evolution, reassortant

## Abstract

Influenza A viruses (IAVs) can infect a wide variety of bird and mammal species. Their genome is characterized by 8 RNA single stranded segments. The low proofreading activity of their polymerases and the genomic reassortment between different IAVs subtypes allow them to continuously evolve, constituting a constant threat to human and animal health. In 2009, a pandemic of an IAV highlighted the importance of the swine host in IAVs adaptation between humans and birds. The swine population and the incidence of swine IAV is constantly growing. In previous studies, despite vaccination, swine IAV growth and evolution were proven in vaccinated and challenged animals. However, how vaccination can drive the evolutionary dynamics of swine IAV after coinfection with two subtypes is poorly studied. In the present study, vaccinated and nonvaccinated pigs were challenged by direct contact with H1N1 and H3N2 independent swine IAVs seeder pigs. Nasal swab samples were daily recovered and broncho-alveolar lavage fluid (BALF) was also collected at necropsy day from each pig for swine IAV detection and whole genome sequencing. In total, 39 swine IAV whole genome sequences were obtained by next generation sequencing from samples collected from both experimental groups. Subsequently, genomic, and evolutionary analyses were carried out to detect both, genomic reassortments and single nucleotide variants (SNV). Regarding the segments found per sample, the simultaneous presence of segments from both subtypes was much lower in vaccinated animals, indicating that the vaccine reduced the likelihood of genomic reassortment events. In relation to swine IAV intra-host diversity, a total of 239 and 74 SNV were detected within H1N1 and H3N2 subtypes, respectively. Different proportions of synonymous and nonsynonymous substitutions were found, indicating that vaccine may be influencing the main mechanism that shape swine IAV evolution, detecting natural, neutral, and purifying selection in the different analyzed scenarios. SNV were detected along the whole swine IAV genome with important nonsynonymous substitutions on polymerases, surface glycoproteins and nonstructural proteins, which may have an impact on virus replication, immune system escaping and virulence of virus, respectively. The present study further emphasized the vast evolutionary capacity of swine IAV, under natural infection and vaccination pressure scenarios.

## 1 Introduction

Influenza A viruses (IAVs) are worldwide distributed pathogens whose natural hosts are many bird and mammal species including humans and pigs. IAVs genome is composed of 8 RNA negative sense strand segments which code for: two polymerases basic (PB1 and PB2) and one polymerase acidic (PA), the hemagglutinin (HA), the nucleoprotein (NP), the neuraminidase (NA), the matrix proteins (M) and the nonstructural proteins (NS1) (Breen et al., 2016). The evolution of IAVs, like other RNA viruses, is described under the quasispecies theory (Domingo et al., 2012; Domingo and Perales, 2019). On one hand, IAVs genome can rapidly accumulate point mutations along the whole genome, because of the low proofreading activity of their polymerases (Chen and Holmes, 2006; Shao et al., 2017), which could confer adaptative advantages to progeny. Also, the evolution of the surface glycoproteins, HA and NA, are constantly under immune pressure if the host has pre-existing immunity against swine IAV. Therefore, both segments, could achieve a new antigenic pattern after genetic diversification, avoiding pre-existing immunity (Carrat and Flahault, 2007). On the other hand, genomic segment reassortments between different IAVs subtypes generate higher levels of variability. This genetic mechanism can occur when a host is simultaneously coinfected by different IAVs and, as a result, new viruses could arise with different host range, antigenicity, and virulence properties. Historically, these reassortment events were responsible for the emergence of important flu pandemics such as the Asian flu in 1957 (H2N2) and the Hong Kong flu of 1968 (H3N2) (Scholtissek et al., 1978; Kawaoka et al., 1989; Belshe, 2005).

In 2009, a new pandemic influenza strain A(H1N1)pdm09 of swine origin quickly spread by human to human contact through more than 30 countries and it has been calculated that caused between 151,700 and 574,400 human deaths associated with the infection (Swine influenza - WOAH - World Organisation for Animal Health, n.d.; Smith et al., 2009). This new strain arose from the reassortment of two circulating swine IAVs, the H1N1 Eurasian “avian-like” (EA) swine strain and a previous circulating human-like H1N2 virus harboring avian, human and swine IAVs segments (Smith et al., 2009; Trifonov et al., 2009). This H1N2 strain arose in 1994 a consequence of reassortment between two strains circulating in pigs, H1N1 and H3N2 (Brown et al., 1998). Lately, in China, a new viral strain H1N1 named as G4 EA, with pandemic and triple reassortant segments, became predominant in the swine population. This strain is capable of infecting humans and, therefore, could pose an imminent pandemic risk (Sun et al., 2020). Hence, pigs play an important role in the generation of new IAVs strains with pandemic potential as they are considered “mixing vessels”, being susceptible to avian, human, and swine IAVs (Ito et al., 1998; Brown, 2000; Ma et al., 2009). Accordingly, pigs could serve as intermediaries in the adaptation of avian IAVs to humans and vice versa (Medina, 2018).

In the last decade, the most circulating subtypes among pig population on European farms have been EA swine H1N1, “human-like” swine H1N2, “human-like” reassortment swine H3N2 and A(H1N1) pdm09 viruses (Simon-Grifé et al., 2011; Torremorell et al., 2012; Kyriakis et al., 2013; Simon et al., 2014; Li and Robertson, 2021). The mortality rate caused by swine IAV in swine is usually low if no major co-infections do occur, but it is a highly contagious disease whose morbidity rate could reach 100% in exposed animals (Er et al., 2014; Li and Robertson, 2021). Both the high seroprevalence and the ever-increasing pig population make reassortment events possibilities being more feasible. Nowadays, biosecurity measures and trivalent vaccine application are the main strategies to avoid disease and reduce swine IAV prevalence (Thacker and Janke, 2008; Salvesen and Whitelaw, 2021), although vaccination is limitedly performed in Europe reaching only 10-20% of the pig population (Reeth et al., 2016). The most used trivalent vaccine against swine IAV in the EU includes H1N1, H3N2, and H1N2 subtypes in its formulation. It reduces virus spread and disease, although it does not generate sterilizing immunity in the host, allowing viral replication (Thacker and Janke, 2008; Ma and Richt, 2010). It is known that swine IAV breakthrough infections are common in pigs because virus evolution favors the escape to the host immunity (Murcia et al., 2012), this situation can promote the circulation of the virus in vaccinated pigs (Chamba Pardo et al., 2018). Previous studies suggest that the application of the trivalent vaccine can influence on the evolutionary dynamics of swine IAV viral populations, being different in vaccinated and nonvaccinated animals (López-Valiñas et al., 2021; López-Valiñas et al., 2022).Thus, the aim of the present study was to evaluate the evolutionary dynamics of swine IAV during a coinfection trial in pigs with EA swine H1N1 and “human-like” swine H3N2 strains in vaccinated and nonvaccinated pigs. Overall, our study retrieved by NGS 39 complete viral quasispecies collected from vaccinated and nonvaccinated pigs, finding different evolutionary patterns, and nonsynonymous substitutions that may play an important role in virulence and the evasion of the swine immune system.

## 2 Material and methodologies

### 2.1 Cells, viruses, and vaccine

Viral titration and production were performed using Madin-Darby Canine Kidney (MDCK, ATCC CCL-34). For cell culture, Dulbecco’s Modified Eagle Medium (DMEM) was used supplemented with fetal bovine serum (FBS) (10%), L-glutamine (1%) and penicillin/streptomycin (1%). The cell culture conditions were 37°C with 5% CO_2_ atmosphere in an incubator.

The A/Swine/Spain/01/2010 H1N1 and the A/Swine/Spain/SF32071/2007 H3N2 viruses were propagated in MDCK monolayer cell cultures at a multiplicity of infection (MOI) of 0.01 and harvested 48 hours later. Virus cell entry was facilitated by 10 µg/mL of porcine trypsin (Sigma-Aldrich, Madrid, Spain) addition. For both virus titrations, serial dilutions in MDCK cells were carried out to calculate the 50% tissue culture infection dose (TCID_50_) (Reed and Muench, 1938).

In the present study, the commercial inactivated swine IAV vaccine (RESPIPORC FLU3, IDT^®^, Dessau-Rosslau, Germany) was used. Vaccine formulation includes the H1N1 (Haselünne/IDT2617/2003), H3N2 (Bakum/IDT1769/2003), and H1N2 (Bakum/1832/2000) strains.

### 2.2 Experiment design

**Figure 1:**
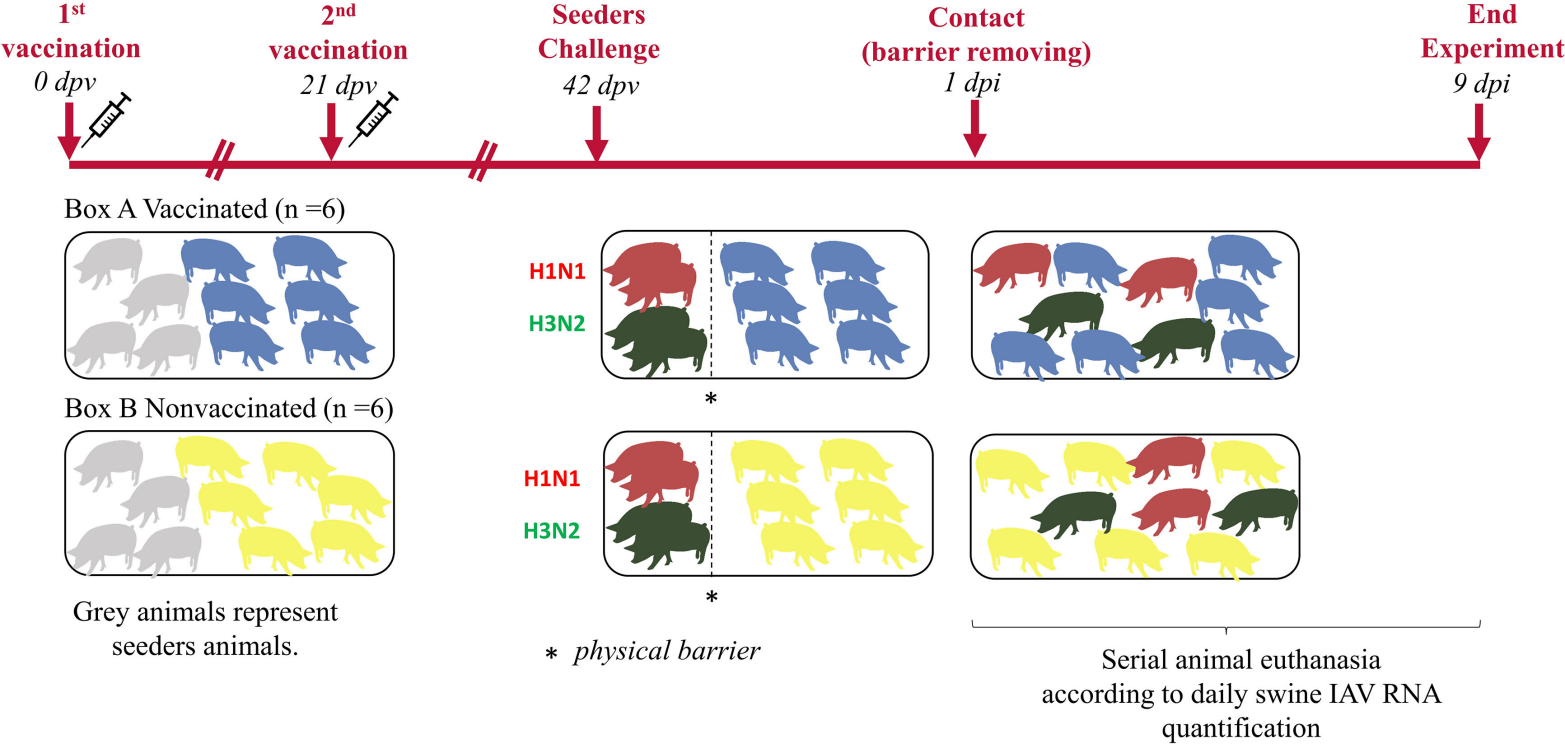
Experimental design and animal distribution. Vaccinated and nonvaccinated pigs are represented in blue and yellow figures, respectively. Seeders animals are represented in grey until the day of their challenge when pigs challenged with H1N1 are represented in red meanwhile those challenged with H3N2 are in green.

Twenty 5-week-old domestic pigs free from swine IAV and its antibodies were selected. Animals were equally distributed in two different boxes of the animal biosafety level 3 (aBSL3) facilities at IRTA-CReSA (**Figure 1**). In one box, seeder animals H1N1 (No. 1 and 2), seeder animals H3N2 (No. 5 and 6) and animals from group A (No. 9 to 14) were allocated. Seeders H1N1 (No. 3 and 4), seeders H3N2 (No. 7 and 8), and animals from group B (No. 15 to 20) were housed in the second box.

After an acclimation period of one week, animals from group A received the first dose of the commercial trivalent vaccine according to manufacturer’s instructions, administering 2 mL intramuscularly in the neck muscle. In parallel, animals from group B received 2 mL of phosphate buffered saline (PBS) in the same manner. Twenty-one days post-vaccination (21 dpv), animals from group A and B, received the second dose of the vaccine and PBS, respectively.

Three weeks after the second vaccination dose (42 dpv), animals 1 to 4 and 5 to 8 were challenged with A/swine/Spain/SF11131/2007 (H1N1) and A/Swine/Spain/SF32071/2007 (H3N2), respectively. For the inoculation, two administration routes, intranasal and endotracheal, were used with a viral concentration of 10^7^ TCID_50_ in a final volume of 2 and 5 mL, respectively. For the intranasal administration, 1 mL of inoculum per nostril was administered with a diffuser device (MAD Nasal, Teleflex, Morrisville, NC, USA). On the other hand, for the endotracheal administration, animals were intubated. For both, a nose snare was used to restrain animals (López-Valiñas et al., 2021). Experimentally inoculated animals were separated from the rest by a physical barrier. One day post inoculation (1 dpi), barriers were lifted to allow direct contact.

After the challenge, animals were daily monitored for rectal temperature, clinical signs, and animal behavior in a blind manner by trained veterinarians. Clinical signs were scored as previously described (Galindo-Cardiel et al., 2013). Moreover, after the first day post-contact (1 dpc), the swine IAV load in nasal swab samples was daily measured by RTq-PCR (explained below). When viral loads started to drop, animals were euthanized.

Nasal swab samples were collected before first and second vaccinations, at day of challenge and daily from 1 dpc to animal euthanasia. Blood samples were collected at vaccinations, challenge, and euthanasia days. From each euthanized animal, lung, nasal turbinate (NT) and broncho-alveolar lavage fluid (BALF) were collected and stored at -80°C. BALF from each animal was collected after filling the right lung with 150 mL of PBS. By last, lung samples (apical, middle and cranial part of diaphragmatic lobes) was fixed by immersion in 10% buffered formalin.

Procedures were approved by the animal ethics committee from the Generalitat de Catalunya, under the project number 10856, following the Spanish and European regulations.

### 2.3 Evaluation of the humoral response against swine IAV

Antibody levels against influenza NP was performed by ID Screen^®^—influenza A Antibody Competition ELISA kit (ID VET, Grabels, France). The inhibition percentages were calculated according to the manufacturer’s instructions where values below 45% were considered positive, greater than 50% negative, and between both values doubtful.

Furthermore, the hemagglutination inhibition (HI) assay was performed in samples collected at day of seeders challenge and each pig euthanasia day as previously described (López-Serrano et al., 2021). All tested serum samples were first treated with RDE II Seiken (Denka Chemicals, Tokyo, Japan) at 37°C during 18 h and later inactivated at 56°C for 1 h. Unspecific hemadsorption inhibitors were removed by adding a volume of 50% chicken red blood cells (RBCs) and later diluted in PBS (1:10). Treated pig sera were two-fold diluted, in v-bottomed 96 well plates, with PBS up to the 1:1024 dilution. Subsequently, 25 µL of each viral isolates used on challenge diluted up to 4 Hemagglutination Units (HAU), were dispensed in each well in parallel and incubated during 1 h at room temperature. Afterward, 25 µL of 0.5% of chicken RBCs were dispensed and again incubated for 1 h at room temperature. The hemagglutination titer of each serum was established as the reciprocal dilution at which inhibition was complete. Titers ≥1/40 were considered protective.

### 2.4 Pathological analyses and immunohistochemistry in lung

The lung parenchyma was macroscopically examined during each animal necropsy. The percentage of lung-affected area was also determined through an image analysis as previously described (Sibila et al., 2014), using ImageJ^®^ software (ImageJ, n.d.).

A sample from each lung section collected in formalin was subsequently dehydrated and embedded in paraffin wax. Later, two 3–5 μm thick sections were cut for both, hematoxylin-eosin (H&E) staining and immunohistochemistry (IHC), for light microscopy examination (Busquets et al., 2010; Sisteré-Oró et al., 2019). For IHC, the two-step polymer method (Envision TM) was conducted using a monoclonal one against influenza A virus (IAV) (Hb65 from the ATCC) and system + HRP-labeled polymer Anti-Mouse (K4001, Dako) as primary and secondary antibodies, respectively (Sabattini et al., 1998). The degree of lung lesion according to the number of airways affected and the amount of immunoreactivity were evaluated, on H&E and IHC examination, respectively, using a previously described semiquantitative scoring system (Detmer et al., 2013).

### 2.5 Swine IAV genome detection

Lung and NT were homogenized in brain heart infusion medium (10% weight/volume) with TissueLyser II (Qiagen, Düsseldorf, Germany). RNA from nasal swab, BALF, lung and NT samples were extracted using the MagAttract 96 Cador Pathogen kit ^®^ (Qiagen, Düsseldorf, Germany) according to manufacturer’s instructions. To detect and quantify swine IAV RNA on each sample a RT-qPCR based on M segment amplification was performed in the Fast7500 equipment (Applied Biosystem) (Spackman et al., 2002). Samples with a cycle threshold (Ct) value under 40 were considered positive whereas no fluorescence detection were considered negative (Spackman et al., 2002; López-Valiñas et al., 2021).

### 2.6 Whole swine IAV genome sequencing

RNA from A/Swine/Spain/01/2010 H1N1 and A/Swine/Spain/SF32071/2007 H3N2 inocula, and RNA extracted from nasal swab and BALF samples with a Ct value lower than 35 were proposed for whole genome sequencing (Zhou et al., 2009; López-Valiñas et al., 2021). First, a whole swine IAV genome amplification was performed using 0.5 μL of each forward MBTuni-12 and reverse MBTuni-13 primers, both at 0.2 μM. Moreover, 0.5 μL of SuperScript^®^ III One-Step RT-PCR System with Platinum™ Taq High Fidelity DNA Polymerase (Thermo Fisher Scientific, Waltham, MA, USA), 12.5 μL of reaction mix included in the kit, 8.5 μL of RNase free water and 2.5 μL of swine IAV RNA. In parallel, a second amplification was performed to enhance biggest swine IAV segments amplification replacing the forward primer by MBTuni12G, keeping the remaining conditions (Lycett et al., 2012). Only samples with whole swine IAV amplification were further selected (Zhou et al., 2009). For sequencing, a multiplexed sequencing library was prepared per sample following the Nextera-XT DNA Library Prep protocol (Illumina^®^, San Diego, CA, USA). Subsequently, the library sequencing was performed using Miseq Reagent Kit v2 in a 150-cycle paired-end run on Miseq Instrument (Illumina^®^, San Diego, CA, USA).

### 2.7 Bioinformatic workflow for quasispecies genomic and evolutionary analysis

In the present study, a bioinformatic workflow was developed to Illumina reads alignment and variant calling, with tools widely used for the study of viral quasispecies (Sobel Leonard et al., 2016; Borges et al., 2018; Cacciabue et al., 2020). First, Illumina adapters were automatically removed. Read quality was checked with FastQC (v 0.11.8) (Andrews, 2010) and low-quality reads (Phread < 30) were trimmed by Trimmomatic (v0.39) (Bolger et al., 2014). To determine swine IAV H1N1 and H3N2 inoculum consensus sequences, reads from both were aligned against the H1N1 (JX908038-45) (Martín-Valls et al., 2014) and H3N2 (HE774666-73) (Mussá et al., 2013) genome sequences using the Burrows-Wheeler alignment (BWA) tool mem function (v0.7.17) (Li and Durbin, 2009). After mapping, unmapped and low quality mapped (< 30) reads were removed from the analysis using Samtools (v.0.39) (Danecek et al., 2021). Moreover, PCR duplicates and reads recalibration were performed with the Picard “MArkDuplicatesSpark” and “BaseRecalibrator” options included in GATK4 (v4.1). Indeed, the sequence depth on each genome position was calculated with “-depth” function included on Samtools (v1.9) and plotted using ggplot2 library in Rstudio (RStudio | Open source & professional software for data science teams - RStudio, n.d.; Wickham, 2016). Subsequently, consensus sequences were generated using consensus option from Bcftools (v.1.9) (Danecek et al., 2021).

All samples were simultaneously aligned, against both inocula consensus, following the previous described workflow. Finally, each sample was again aligned, using only as a reference the segments that were present on each specific sample. In all samples, SNV reported because of misalignment reads due to the high percentage of similarity between H1N1 and H3N2 sequences, were parsed and eliminated using R (v.4.1.2) (RStudio | Open source & professional software for data science teams - RStudio, n.d.).

All variants found along the whole genome were noted using LoFreq software with default parameters (Wilm et al., 2012). Moreover, the effect of each substitution noted on variants was predicted with SnpEff software (v.4.3) (Cingolani et al., 2012). Previously, a database for both subtypes was created with “build–gtf22” function, using the previously annotated genomes (Mussá et al., 2013; Martín-Valls et al., 2014). The following requirements were considered to call a single nucleotide variant (SNV); p value <0.01 and at least 50 and 10 reads of depth and alternative base count respectively (López-Valiñas et al., 2021). Finally, the nucleotide diversity (π) per genomic segment per sample were calculated with SNPGenie software (Nelson et al., 2015).

### 2.8 Protein structure representation

Allocations of nonsynonymous substitutions with an allele frequency greater than 5% were pointed in each swine IAV protein. Lolliplot proteins representation was done with the trackViewer package from Bioconductor (Ou and Zhu, 2019). All protein domain delimitation was inferred as previously reported (Sriwilaijaroen and Suzuki, 2012; Hu et al., 2014; McAuley et al., 2019). Besides, HA and NA 3D protein representations were also performed with the PyMOL Molecular Graphics System (v.4.6).

### 2.9 Statistical analysis

The T-test was used to compare the distribution of the antibody levels against influenza NP on the day of the challenge, and Ct values from 4 to 9 dpi, between vaccinated and nonvaccinated animals. The Chi-squared test was used to study differences in proportions between the numbers of genomic segments detected and synonymous and nonsynonymous percentages in vaccinated and nonvaccinated pigs. Subsequently, the Bonferroni correction was applied. Finally, an analysis of variance (ANOVA) and subsequent *post-hoc* Kruskal-Wallis test by rank, were applied to compare π means between vaccinated and nonvaccinated animals on different dpi and in different segments.

## 3 Results

### 3.1 Humoral response against swine IAV

**Figure 2:**
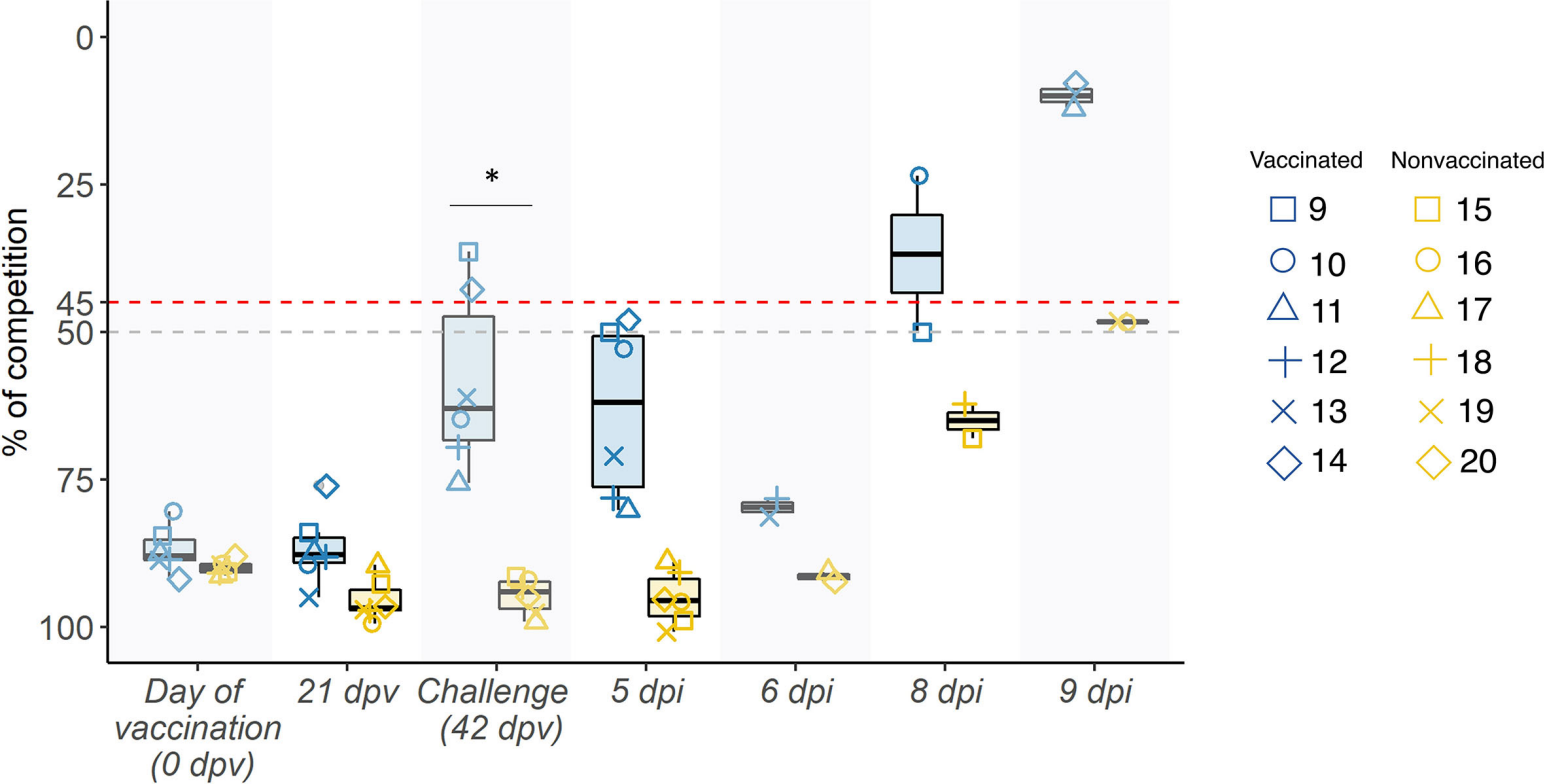
Swine IAV NP antibody kinetics in serum samples represented in boxplots. The percentage of competition is expressed in the ordinate axis whereas the sampling day is indicated on the abscissas one. Blue boxplots show samples collected from vaccinated animals, whereas the yellow ones represent nonvaccinated ones, and the whiskers show quartile variability. Different shapes illustrate the ID number of each pig. Values above the red line (<45%), below the grey line (>50%) and between both lines are considered positive, negative, and doubtful respectively. *P < 0.05.

Competition percentage means of antibodies against NP were greater at all sampling days in vaccinated animals. From day of challenge until the end of the experiment, difference between groups were significant (*p=* 0.001881; *t-test*). At day of challenge, 2 out of 6 serum samples collected from vaccinated animals were considered positive, but in the whole group an increase in the antibody titers has been observed, indicating that an immune response has taken place (**Figure 2**).

Regarding HI titers against strains used for the inoculation at the day of challenge showed no positive titers against H1N1 subtype in vaccinated pigs, while all sera had positive titers against H3N2 subtype (**Table 1**), even though the percentage of amino acid identity is similar between the vaccine viruses and the challenge viruses (96,47% for H3 and 95,76% for H1). Additionally, from the 50 positions involved in antigenic sites, only position 204 (antigenic site Ca1) shows different amino acids between vaccine and virus challenge (D and V, respectively). On the day of euthanasia, titers against H1N1 subtype were only detected in vaccinated animals, specifically in 3 out of 6 animals. On the other hand, positive titers against H3N2 were observed in all samples collected from vaccinated animals, whereas were only detected in nonvaccinated pig sera at 8 and 9 dpi with low titers.

**Table 1:** HI titers of sera samples collected from vaccinated and nonvaccinated animals against H1N1 (left) and H3N2 (right) strains used on the challenge.

		HI titers against H1N1					HI titers against H3N2				
Group	Pig ID	42 dpv	Euthanasia Day				42 dpv	Euthanasia Day			
			6 dpi	7 dpi	8 dpi	9 dpi		6 dpi	7 dpi	8 dpi	9 dpi
Vaccinated Animals	*9*	*0*			*0*		*160*			*80*	
	*10*	*0*			*20*		*80*			*80*	
	*11*	*0*			*320*		*40*			*80*	
	*12*	*0*	*0*				*80*	*40*			
	*13*	*0*	*0*				*80*	*160*			
	*14*	*0*				*160*	*160*				*320*
Nonvaccinated Animals	*15*	0			0		0			10	
	*16*	0				0	0				40
	*17*	0	0				0	0			
	*18*	0			0		40			40	
	*19*	0				0	0				80
	*20*	*0*	*0*				*0*	*0*			

Sampling days are specified on the top of each table. Empty cells indicate no sampling. Titers greater than 40 are considered positive.

### 3.2 Swine IAV genome was detected in both experimental groups after seeder contact

**Figure 3:**
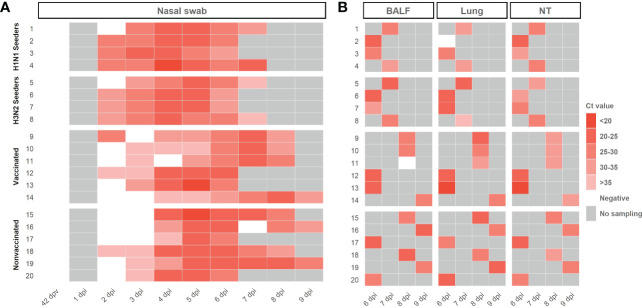
Swine IAV genome detection in samples collected from H1N1 and H3N2 seeders, vaccinated and nonvaccinated pigs **(A)** Daily swine IAV RNA detection in samples collected from nasal swabs. **(B)** Swine IAV genome detection in BALF, lung, and nasal turbinates collected on the euthanasia day. The Ct values are represented as a heat map where the maximum intense red colors represent higher viral loads and vice versa. RT-qPCR CT values greater than 40 were considered negative, represented in white. No sampling collected was represented as grey. The ID of each animal and the day at which each sample was collected are represented in ordinate and abscissa axes, respectively.

Swine IAV RNA was detected in nasal swab samples collected from seeders, vaccinated and nonvaccinated animals, from 2 dpi until the end of the experiment (**Figure 3A**; **Supplementary Table S1**). swine IAV was not detected in any nasal swab sample collected before the challenge (42 dpv) (**Figure 3A**). As average, lower Ct values were detected in samples collected from nonvaccinated animals in comparison with vaccinated ones, being this difference significant at 6 dpi (t-test; *p=0.0186*) with a mean Ct value of 24,23 ± 1,33 and 27,68 ± 2,51, respectively. In total, 32 out of 39 and 31 out of 40 nasal swab samples analyzed from vaccinated and nonvaccinated animals, respectively, were RT-qPCR swine IAV positive. Besides, 23 vaccinated and 26 nonvaccinated samples obtained Ct values lower than 35.

Regarding BALF, lung, and nasal turbinate samples, swine IAV was detected in all samples except in BALF from vaccinated animal No. 11 and lung from animal No. 2. No significative differences were observed between groups, showing similar mean Ct values (**Figure 3B**; **Supplementary Table S1**). All positive BALF samples had a Ct value lower than 35.

### 3.3 Pathological findings

On average, the lung affected area was greater in vaccinated animals, although statistical differences were not found. Vaccinated animals No. 9 and No. 10 had higher percentage of pneumonic lesions, 17.89 and 31.89% respectively. The remaining pigs did not exceed 6% of lung affected area (**Table 2**).

**Table 2:** Lung pathological results according to the percentage of total lung-affected area, and the semi-quantitative scoring for the degree of airways affectation and amount of immunoreactivity.

Group	Pig ID	Euthanasia day	Lung affected area (%)	Histopathological score	Immunohistochemical score
*Vaccinated pigs*	*9*	8 dpi	17.96	3	++
	*10*	8 dpi	31.89	3	++
	*11*	8 dpi	5.52	2.5	–
	*12*	6 dpi	3.19	3	++
	*13*	6 dpi	0.19	2.5	++
	*14*	9 dpi	6.09	2.5	++
		*mean*	10.81	2.75	
*Nonvaccinated pigs*	*15*	8 dpi	NO DATA	1.5	++
	*16*	9 dpi	4.86	3	+
	*17*	6 dpi	0.48	2	++
	*18*	8 dpi	5.43	2.5	++
	*19*	9 dpi	5.65	3	+
	*20*	6 dpi	0.62	1.5	++
		*mean*	3.41	2.25	

Histopathological score: absence (0), few inflammatory cells isolated (0.5), a localized cluster of inflammatory cells (1), several clusters of inflammatory cells (1.5–2), severely several (2.5), and many airways affected (3). Moreover, minimal (1.5), mild (2) interstitial infiltrate, and plus moderate interstitial and alveolar infiltrate (2.5–3). Immunohistochemical score: absence (-), low (+), scattered (++), moderate (+++), and abundant (++++) amount of immunoreactivity.

All pigs, irrespectively from their vaccination status, developed a mild-to-severe broncho-interstitial pneumonia, with scores ranging from 1.5 to 3. In vaccinated pigs, three animals had the highest histopathological scoring, whereas the remaining ones obtained the second highest score value, with 2.5. By contrast, an average lower microscopic lesion scores were detected in nonvaccinated pigs. Regarding immunohistochemistry, all lungs had scattered amount of immunoreactivity except nonvaccinated animals No. 16 and 19, with low amounts, and vaccinated pig No. 11, with no immunoreactivity.

### 3.4 Whole genome sequencing of inocula, nasal swab, and BALF collected from vaccinated and nonvaccinated animals

The whole swine IAV genome from inocula A/swine/Spain/SF11131/2007 (H1N1) and A/Swine/Spain/SF32071/2007 (H3N2) was sequenced (**Figure 3A**). From samples collected after contact challenge, 39 swine IAV genomes were obtained, 20 from vaccinated animals and 19 from nonvaccinated ones (**Supplementary Table S1**). After sequenced samples alignment, a total of 2.467.438 reads of swine IAV were obtained, 1.347.544 from vaccinated samples and 1.119.899 from nonvaccinated ones; 67% of these reads matched against the H1N1 genome whereas the remaining 33% did it against H3N2 subtype (**Supplementary Table S2**).

**Figure 4:**
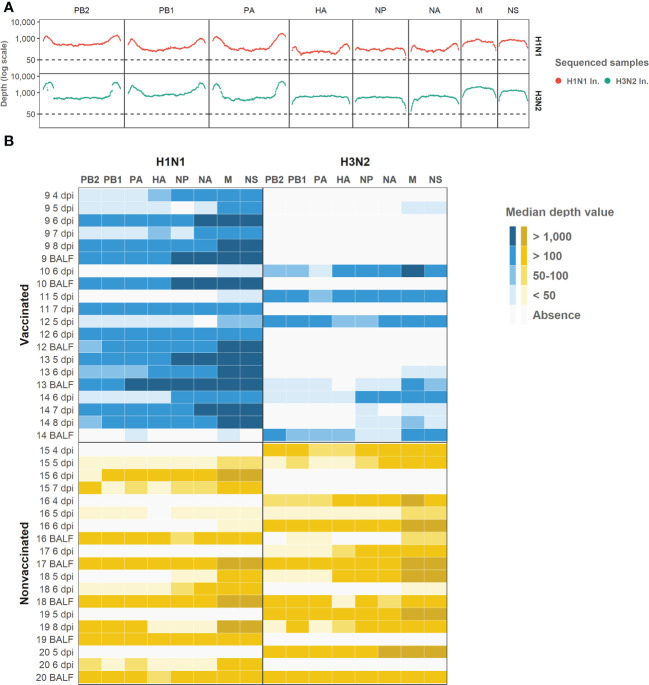
Illumina sequencing reads aligned against each subtype genomic profile. **(A)** Illumina sequencing profile of swine IAV H1N1 (plotted in red) and H3N2 (plotted in green) used as inocula. In the x axis each position per genomic segment is indicated whereas the depth of read is indicated in y axis in the logarithm scale. **(B)** Heat map of the median depth value obtained in all sequenced samples from vaccinated and nonvaccinated animals per H1N1 (left) and H3N2 (right) genomic segments. Genomic segments per subtype and samples are indicated in the x and y axes, respectively. The name of each sequenced sample indicates the origin and the day of its collection. BALF samples were collected at each pig necropsy.

Regarding the depth per position and the coverage (**Figure 4**), 74.8% of H1N1 swine IAV whole genome positions sequenced were represented with a depth of more than 50 reads. On the other hand, the 6% of the H3N2 position sequenced were also represented by more than 50 reads. Those positions were selected for further variant analysis. In general, NS and M segments obtained the greatest median depth values (**Figure 4**), meanwhile, the biggest segments PA, PB1 and PB2 got the lowest values.

A total of 1947 out of 2544 called variants were deleted because of misalignment reads due to the high percentage of similarity between H1N1 and H3N2 sequences (**Supplementary Table S3**). Hence, 597 SNVs were further analyzed.

### 3.5 Determination of H1N1 and H3N2 genomic segment per sample

**Figure 5:**
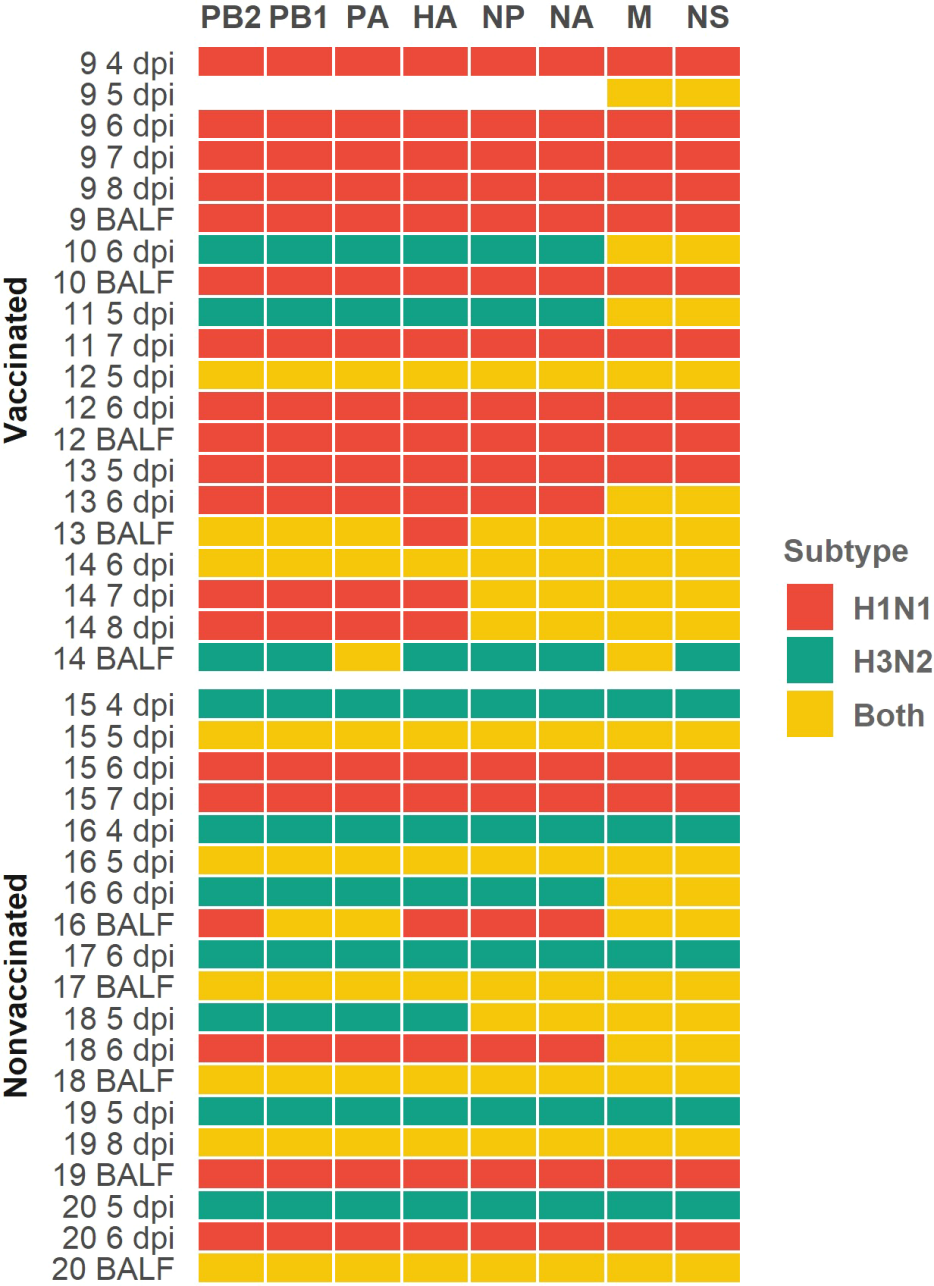
Profile subtype identification per genomic segment in each sequenced sample heatmap. H1N1 subtype segments are indicated in red cells, H3N2 ones are indicated in green cells and yellow ones indicate that both subtypes were simultaneously found. Genomic segments and samples collected from vaccinated and nonvaccinated animals are represented on the x and y axes respectively. The number of each sequenced sample indicate the animal from which each sample was collected. Sample collection day are also indicated as dpi in nasal swab samples; BALF samples were collected on the day of necropsy.

In relation to the subtype segments profile per sample, in vaccinated animals we obtained 95 H1N1 segments, 18 H3N2 segments, and 41 segments where both subtypes were simultaneously found (**Figure 5**). Meanwhile, in nonvaccinated pigs, 42 H1N1, 50 H3N2, and 60 segments with both subtypes were found (**Figure 5**). The distribution of H1N1 and H3N2 segments was statistically different between groups (*P* = 0.0002, *P* = 0.000018, respectively; chi-squared), being greater the number of H1N1 segments in vaccinated animals and greater the number of H3N2 segments in nonvaccinated ones. Regarding the number of segments in which sequences from both subtypes were found, in vaccinated animals this number was lower than that of nonvaccinated ones, although this difference was not significant (*P* = 0.0587; chi-squared).

### 3.6 Variants detected in inocula and their evolution in samples sequenced from infected pigs

From both inocula, 14 and 9 SNV were detected from H1N1 and H3N2 sequences, respectively (**Table 3**). For H1N1 sequences, 8 out of 14 substitutions found were synonymous, whereas for H3N2 ones all SNV detected were nonsynonymous. Furthermore, most of the SNV detected in H1N1 sequences (12 out of 14) exceed the 5% of allele frequency whereas none of them did it for the H3N2 inoculum (**Table 3**).

**Table 3:** SNV detected in H1N1 and H3N2 inoculum samples.

	Gene	Depth of read	Nucleotide change		Alt. Base count	Allele frequency	Effect on variant	Aminoacidic change	
			position	ref. → alt.				position	ref. → alt.
**Inoculum H1N1**	NS1	860	323	A → C	107	12.44	nsyn	108	K → T
		731	631	G → A	228	31.19	nsyn	211	G → R
	M1	681	240	C → T	435	63.88	syn	80	V → V
		702	261	T → C	23	3.28	syn	87	N → N
		961	396	C → T	14	1.46	syn	132	Y → Y
	M2	603	896	R → A	312	51.74	nsyn	70	X → K
		603	896	R → G	291	48.26	nsyn	70	X → E
	NA	274	150	T → A	56	20.44	nsyn	50	N → K
	PA	189	1055	A → G	10	5.29	nsyn	352	E → G
	PB1	244	1146	T → C	52	21.31	syn	382	N → N
		256	1185	C → T	48	18.75	syn	395	L → L
		322	1254	Y → C	132	40.99	syn	418	V → V
	PB2	405	1054	T → C	98	24.2	syn	352	L → L
		990	168	G → A	224	22.63	syn	56	P → P
**Inoculum H3N2**	HA	589	1111	C → T	15	2.55	nsyn	371	H → Y
	PA	369	1046	A → G	15	4.06	nsyn	349	E → G
		574	290	C → T	13	2.26	nsyn	97	T → I
		4042	2047	C → A	161	3.98	nsyn	683	L → I
		760	1852	G → A	10	1.32	nsyn	618	V → I
	PB1	827	1308	C → T	12	1.45	nsyn	436	Y → Y
	PB2	3026	153	G → A	33	1.09	nsyn	51	M → I
		440	395	C → T	15	3.41	nsyn	132	P → L
		1044	2033	A → C	11	1.05	nsyn	678	D → A

ref. (reference), alt. (alternative), nsyn (Nonsynonymous) and syn (Synonymous).

**Figure 6:**
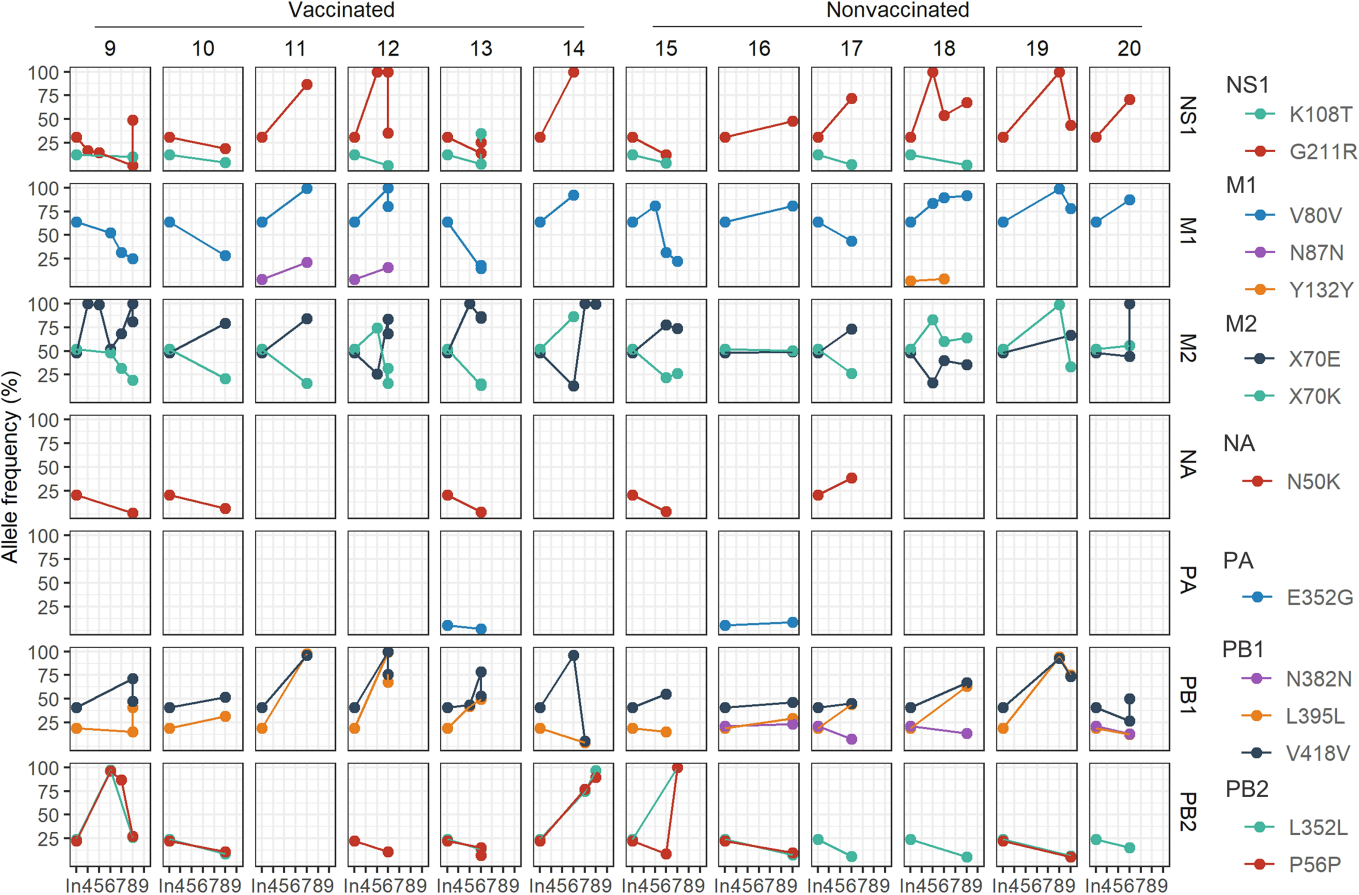
Evolution of the allele frequencies of nonsynonymous substitutions detected in H1N1 inoculum and later in samples collected from both experimental groups. The day of variant detection and the allele frequency percentage is represented in ordinate and abscissa axes, respectively. The “In.” in x axis indicates the percentage obtained in the inoculum, whereas 4 to 9 numbers indicate the days post-inoculation. In each column, the different animals are represented while in each row NS1, M1, M2, NA, PA, PB1, and PB2 proteins are indicated. Each mutation is plotted in different colors.

These SNV were later detected in samples collected from vaccinated and nonvaccinated animals in the HN1 inoculum (**Figure 6**). In most of the sequenced samples, the SNVs G211R (NS1), V80V (M1), X70E (M2), L395L, and V418 (PB1) increased their allele frequency over time. On the other hand, allele frequencies of NS1 K108T, M1 N87N and Y132Y, NA N50K, PA E325G, PB1 N382N, and PB2 substitutions tended to decrease until they were no longer detected, with some exceptions (**Figure 6**). Notably, all SNV detected in H3N2 inoculum were no later detected in sequenced samples.

### 3.7 *De novo* SNVs identification; synonymous and nonsynonymous proportion and allocation along swine IAV genome segments

**Figure 7:**
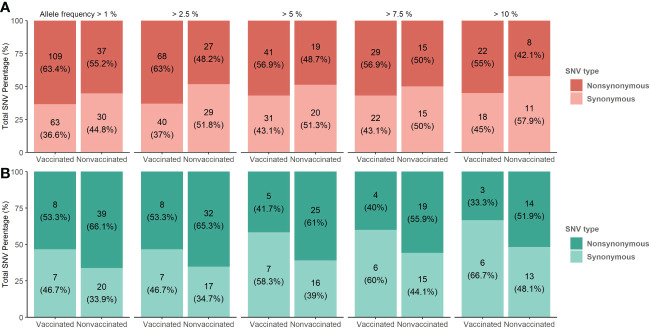
*De novo* synonymous and nonsynonymous SNV proportion bars in samples from vaccinated and nonvaccinated animals at different allele frequencies. **(A)** SNV proportions found in the H1N1 subtype are represented in red bars **(B)** SNV proportions found in the H3N2 subtype are represented in green bars. The total number of SNV and its percentages are indicated in each bar. Dark and soft colors represent nonsynonymous and synonymous SNV, respectively.

**Figure 8:**
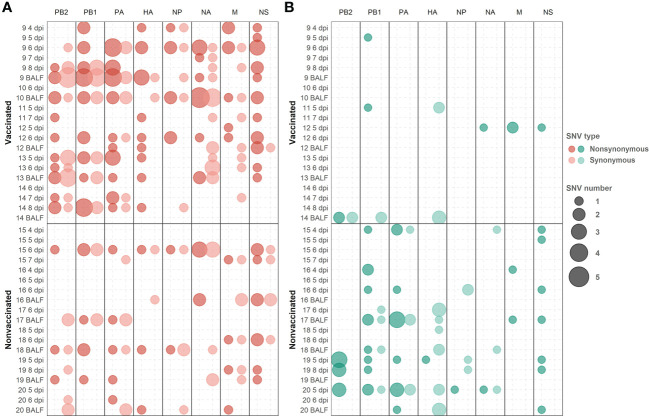
*De novo* synonymous and nonsynonymous SNV with an allele frequency greater than 1% allocation along swine IAV genome. **(A)** SNVs found in the H1N1 subtype are represented in red circles. **(B)** SNV found in the H3N2 subtype are indicated in green circles. Nonsynonymous and synonymous substitutions are represented in dark and light colors, respectively. Circle size indicates the total number of SNV found per genomic segment and samples, in abscissa and ordinate axes respectively.

A total of 313 SNVs were found in all analyzed samples, 187 in vaccinated animals and 126 in nonvaccinated ones (**Figures 7** and **8**). In vaccinated pigs, 172 were found in H1N1 and 15 in H3N2 subtypes, meanwhile, in nonvaccinated animals 67 and 59 were identified per subtype, respectively. Regarding SNVs per subtype, 239 were found in H1N1 and 74 in H3N2 (**Figure 7**).

The proportions of the total synonymous and nonsynonymous *de novo* SNV were studied from 1 to 10% allele frequency in sequenced samples from vaccinated and nonvaccinated animals (**Figure 7**). Regarding the H1N1 subtype, the proportion of nonsynonymous variants was greater than synonymous ones for all allele frequencies analyzed in both experimental groups. This difference was greater in vaccinated animals although no statistical differences between groups were found (**Figure 7A**). In contrast, these proportions were very close to 50% in nonvaccinated animals, being this trend consolidated throughout the allele frequencies analyzed. For H3N2, the proportion of nonsynonymous SNVs was greater in both scenarios, specifically in samples from nonvaccinated animals (**Figure 7B**).

Considering those variants whose allele frequency was greater than 1%, SNV were allocated along the whole genome of both studied subtypes (**Figure 8**; **Supplementary Table S4**). In general, more substitutions were found in samples from vaccinated animals for the H1N1 subtype (**Figure 8A**). Specifically, a total of 54 *de novo* nonsynonymous variants were found in polymerase segments in samples from vaccinated animals, while only 14 were reported in nonvaccinated pigs. In the HA and NA segments, 12 and 13 nonsynonymous substitutions were reported from vaccinated animals while only 3 and 5 were found in the nonvaccinated ones, respectively. Regarding the NS, a total of 17 nonsynonymous variants were identified in vaccinated animals and 9 in nonvaccinated pigs. Lastly, segments in which the least nonsynonymous substitutions appeared were in the M and the NP proteins, with 6 and 7 in vaccinated and 4 and 2 in nonvaccinated pigs, respectively (**Figure 8A**). Regarding SNV found in the H3N2 subtype, only 8 nonsynonymous substitutions were found in vaccinated animals, 2 in PB2, 2 in PB1, 2 in M, and only 1 in NA and NS. Nevertheless, no nonsynonymous substitutions were reported in PA, HA, and NP (**Figure 8B**). On the other hand, in the nonvaccinated group, the largest number of nonsynonymous variants were also found in the polymerase segments, 11 in PA, 11 in PB1 and 8 in PB2. In the remaining segments, 4 nonsynonymous variants were detected in NS, 2 in M, and only one was reported in HA, NA, and NP (**Figure 8B**).

### 3.8 *De novo* nonsynonymous SNV with an allele frequency greater than 5% are allocated on protein domains

**Figure 9:**
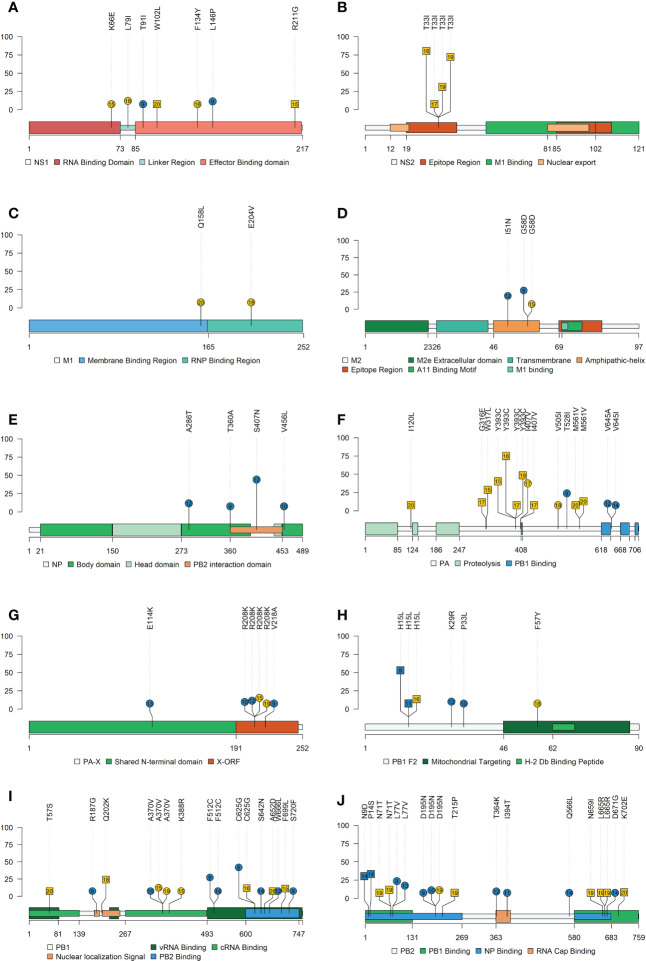
Lolliplot representation of nonsynonymous SNV allocation on swine IAV proteins. Substitutions found in NS1 **(A)**, NS2 **(B)**, M1 **(C)**, M2 **(D)**, NP **(E)**, PA **(F)**, PA-X **(G)**, PB2-F2 **(H)**, PB1 **(I)** and PB2 **(J)** proteins with an allele frequency greater than 5%. In the ordinate axis, the allele frequency of each substitution is indicated. Substitutions noted in blue and yellow show substitutions found in vaccinated and nonvaccinated animals. Besides, the circle shape indicates that substitution was reported in H1N1 subtype, whereas substitutions found in H3N2 are represented by square shapes. The number inside each shape indicates the pig ID of each reported substitution. Figure legends indicate the most important domains of each protein.

**Figure 10:**
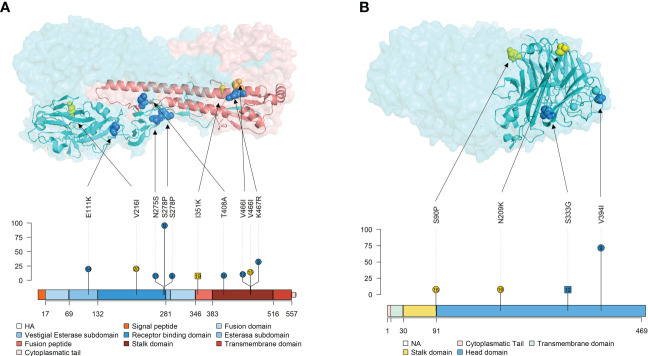
Localization of nonsynonymous substitutions, with an allele frequency greater than 5%, found in HA and NA surface glycoproteins on 3D and lolliplot protein representation. **(A)** HA trimer structure (PDB accession no. 3LZG (Xu et al., 2010)) and domains representation by lolliplot. HA1 and HA2 domains are represented in different tones of blues and reds respectively. **(B)** NA tetramer structure (PDB accession no. 4B7Q (van der Vries et al., 2012)) and domains representation by lolliplot. In both 3D representations, substitutions are highlighted in blue, yellow and orange if it were found in samples from vaccinated, nonvaccinated and both, respectively. In the lolliplot representation, substitutions are plotted in blue and yellow (vaccinated or nonvaccinated groups, respectively), circle and squared shapes (H1N1 and H3N2 subtypes, respectively) and number on shapes indicate the animal in which each substitution was found. In the ordinate axis, the substitution allele frequency is indicated. The different surface glycoprotein domains are indicated in lolliplot legends.

For the H1N1 subtype, the following nonsynonymous SNV exceeding the 5% of allele frequency were reported in vaccinated animals: NS1 (T91I and L146P), ion channel (M2) (I51N and G58D), NA (V394I), NP(A286T, T360A, S407N, and V456L), HA (E111K, N275S, S278P, T408A, V466I, and K467R), PA (T528I, V645A, and V645I), PA-X (E114K, R208K, and V218A), PB1 (R187G, A370V, F512C, C625G, S642N, W666L, and S720F), PB1-F2 (K29R and P33L), and PB2 (L77V, D195N, T364K, I394T, Q566L, and D671G). Meanwhile, in nonvaccinated animals, substitutions were allocated in NS1 (K66E, L79I, and F137Y), M2 (G58D), matrix protein (M1) (Q158L and E204V), NA (S90P and N209K), HA (V216I and V466I), PA (I407V and V505I), PA-X (R208K), PB1 (A370V and K388R), PB1-F2 (F57Y), and PB2 (D195N) (**Figures 9** and **10**).

Regarding substitutions found in the H3N2 subtype in vaccinated pigs, SNVs were noted in PB2 (N9D and P14S), PB1-F2 (H15L), and NA (S333G). On the other hand, in nonvaccinated animals substitutions found were allocated in NS1 (W102L and R211G), nuclear export protein (NS2) (T33I), HA (I351K), PA (I120L, G316E, W317L, S388G, Y393C, I407V and M561V), PB1 (T57S, Q202K. C625G, A652D, and F699L), PB1-F2 (H15L), and PB2 (N71T, T215P, N659I, L665R, and K702E) (**Figures 9** and **10**). All the substitutions found in the present study, including those with lower allele frequency, were noted in **Supplementary Table 4**.

### 3.9 Nucleotide diversity

**Figure 11:**
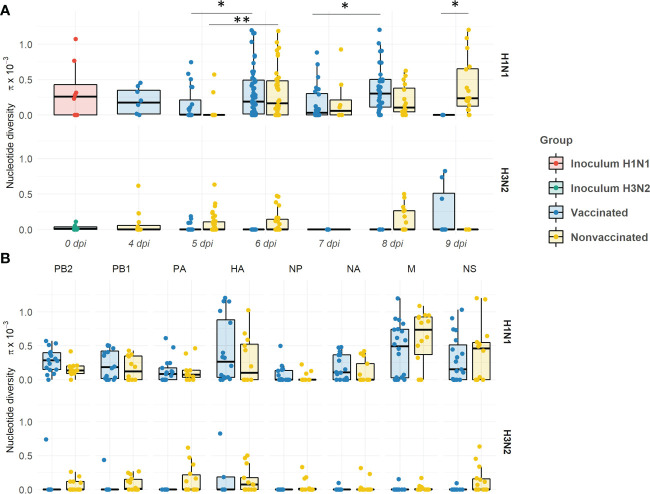
Evolution of the nucleotide diversity (π) in H1N1 and H3N2 viral quasispecies in vaccinated and nonvaccinated animals over time **(A)** and per genomic segment **(B)**. Dots in the boxplot indicates π in each genomic segment. Boxplots indicate means, lower and upper quartile, and standard deviation. The nucleotide diversity of H1N1 and H3N2 inocula, and samples collected from vaccinated and nonvaccinated animals are represented in red, green, blue, and yellow respectively. *P < 0.05 and **P < 0.001..

Within the H1N1 subtype, the nucleotide diversity (π) values fluctuated over time in both experimental groups (**Figure 11A**). The π significantly increased in vaccinated animals from 5 to 6 dpi, and from 7 to 8 dpi (*P = 0.03134 and 0.003948*, respectively; *Kruskal-wallis*). However, π decreased at 5 and 7 dpi in comparison with each previous day, although this decrease is not significant. Within the H3N2 subtype, the π only significantly increased from 5 to 6 dpi (*P = 0.000221; Kruskal-wallis*). In general, π means were greater in vaccinated animals. However, the π mean in nonvaccinated animals was slightly higher at 7 dpi, and significantly higher at 9 dpi (*P = 0.04804; Kruskal-wallis*). On the other hand, within H3N2 subtype, higher levels of π were observed in samples from nonvaccinated pigs at 5, 6, and 8 dpi, whereas in vaccinated animals the highest levels of π were observed at 9 dpi.

Regarding π per genomic segment per group, the diversity was higher within the H1N1 subtype in comparison with the H3N2 one (**Figure 11B**). A greater π mean was observed in all segments of the H1N1 subtype in vaccinated animals, except in M and NS segments. The highest π was found in the HA. No significant differences per genomic segment and group were observed.

## 4 Discussion

In this work, a commercial trivalent vaccine was applied to immunize animals from the vaccinated group. On the day of the seeders contact, higher levels of antibodies against NP swine IAV protein were detected in vaccinated animals, with the highest percentage of competition in pig No. 9. Regarding HI activity, before the challenge, only sera from vaccinated animals had hemagglutinating titers against H3N2 subtype. On the contrary, no sera had HI activity against H1N1 at the time of contact with seeders, although it was subsequently detected in vaccinated pigs No. 10, 11, and 12 at necropsy day. This is a common effect that the vaccine provides a priming effect, and a boost is observed when the virus challenge is inoculated. This has been widely described for influenza and other viruses (Ganges et al., 2008; Díaz et al., 2013; Tarradas et al., 2014; Kim et al., 2021; Pliasas et al., 2022). Thus, we consider that the vaccine against H1N1 has a limited specific immune effect. According to swine IAV detection, the virus was detected in seeders, vaccinated, and nonvaccinated animals. Hence, the reproduction of a direct contact infection with swine IAV was achieved. Besides, the Ct value means indicated that, from 4 to 9 dpi, the viral loads were greater in nonvaccinated animals, especially at 6 dpi when mean Ct value is 3,45 times lower than vaccinated ones. Therefore, the vaccine had an effect in terms of modestly reducing viral seeding, so selective pressure on virus genome is feasible to study due to humoral immunity caused by vaccination (Murcia et al., 2012; López-Valiñas et al., 2021; López-Valiñas et al., 2022).

On the contrary, the effect of the vaccine was not as effective regarding the pathological findings, as the percentage of lung affected area and histopathological score means were greater in vaccinated animals, meanwhile similar results between groups were observed in relation to the immunohistochemical score. In vaccinated animals, animals No. 9 and 10 had the highest percentage of lung lesions with 17.96 and 31.89%, respectively. The remaining animals had similar values to pigs from the nonvaccinated group. Hence, pigs No. 9 and 10 seemed to be considered outliers. It cannot be ruled out that those animals may be suffering from vaccine-associated enhanced respiratory disease (VAERD), that has been previously described for influenza immunization (Vincent et al., 2008; Gauger et al., 2011; Kimble et al., 2022) This adverse effect is produced when the inactivated vaccine and the infection strains are of the same subtypes but with heterologous antigenicity. In consequence, and overall, the vaccine worked in terms of viral shedding reduction, but it did not avoid the swine IAV clinical and pathological manifestations.

Herein, 41 swine IAV quasispecies were analyzed by NGS, including 2 inocula, 20 samples from vaccinated animals, and 19 from nonvaccinated ones. In samples from vaccinated animals, H1N1 segments alone were more widely detected in each genomic profile (95 times), meanwhile, H3N2 segments alone were barely detected (18 times). On the contrary, in the nonvaccinated group, the presence of both segments simultaneously found was the most frequent profile detected, followed by H3N2 alone. These results indicate that vaccination modified the proportion of viral populations, favoring the dominance of the H1N1 subtype, probably because of the absence of strong detectable specific immunity against this subtype. Such effect seemed to decrease the probability of two subtypes simultaneously coexistence, therefore resulting in a decreased probability of genomic reassortment. Contrary, because of the dominant presence of the H1N1 subtype in vaccinated pigs, a greater increase in swine IAV nucleotide diversity, and the greatest number of *de novo* SNVs (total of 170) were found. The reduction in the presence of H3N2 segments is also reflected in the number of *de novo* SNV in this subtype (total of 15). Thus, in this scenario, the application of the vaccine caused the H1N1 swine IAV prevailing over H3N2, which has an impact on its evolution. On one hand, the probability of reassortments was reduced in vaccinated pigs, as has been previously reported (Li et al., 2022). On the other hand, swine IAV H1N1 genetic variability increased in this group of animals. In the long term, this could allow viral genomic diversification and, in consequence, increasing the likelihood of immune escape mutants generation, as previously reported in IAV (Zharikova et al., 2005; Sitaras et al., 2014; Yasuhara et al., 2017; Park et al., 2020; Sitaras et al., 2020; Xu et al., 2022). The amino acid identity percentage matrix among HAs and NAs from both, challenge, and vaccine strains, are available in the **Supplementary Table S5**.

Regarding the SNV and the nucleotide diversity found in both inocula, the H1N1 strain used resulted more genetically diverse than the H3N2 one, since more SNVs were reported, and they were also represented with a higher allele frequency. Substitutions found in the H1N1 inoculum were also detected in samples collected from both experimental groups, although SNVs allele frequencies were later decreasing over time, or they were no longer detected. However, there were also some substitutions such as, PB2 P56P, PB1 V418V, M1 V80V, M2 X70K/E, and NS1 G211R, whose allele frequencies increased in some samples over time. These variants continue belonging to the viral population despite having suffered a bottleneck effect, as showed in **Figure 11** where π decreased in viral samples recovered from infected pigs in comparison with the inoculum. As opposed to that observed for H3N2, the lack of a strong detectable neutralizing activity against H1N1 in vaccinated animals may support the higher genomic diversification of H1N1 subtype. Considering the quasispecies theory, these mutants could display a beneficial effect on virus fitness, since they were present in the initial viral population mutant spectrum, and they remained after a selection event, meanwhile the remaining disappeared. On the other hand, all SNVs detected in the inoculum H3N2 viral quasispecies spectrum were no longer detected in virus collected from any pig samples. All variants detected in both inocula were probably generated, and maintained over time, during virus passages in MDCK cells, because of virus adaptation to cells. Thus, the loss of these variants is probably due to the re-adaptation of the virus to its natural host.

The proportion of nonsynonymous and synonymous *de novo* SNV found may indicate that the viral quasispecies evolution is under selective pressure (Sobel Leonard et al., 2016). In the H1N1 subtype, in vaccinated animals the proportion is greater, indicating that viral evolution may be under positive selection in this scenario (Fitch et al., 1991; Li et al., 2011). However, neutral selection may be acting in nonvaccinated animals, as the same proportion of SNVs was detected (Frost et al., 2018). Therefore, in both scenarios, genetic diversification is occurring. Many of the new mutations generated in H1N1 subtype during the experimental trial probably play a neutral role in viral fitness, such as the synonymous variants, while others are generated because of the viral readaptation from cell culture to its natural host. However, a higher proportion of nonsynonymous substitutions were generated in vaccinated animals, which could be indicating that, additionally, the humoral immune pressure is acting as an evolutionary selection force. By contrast, in the H3N2 subtype, in nonvaccinated animals, the highest proportion of nonsynonymous mutations may be indicating that the virus is also under positive selection. Similar results have been previously found with the same strain; therefore, virions may be poorly adapted to swine hosts, probably because of the readaptation from cell culture (Sobel Leonard et al., 2016; López-Valiñas et al., 2022). In vaccinated animals, as previously mentioned, the viral shedding was greatly reduced and, consequently, not enough variants have been detected. Therefore, the main force that drives viral evolution in this scenario could not be inferred.

In this study, the rapid evolutionary capacity and plasticity of swine IAV are again evidenced (Murcia et al., 2012; Diaz et al., 2013; Diaz et al., 2015; López-Valiñas et al., 2021; López-Valiñas et al., 2022), since 313 *de novo* synonymous and nonsynonymous SNV were reported from all the quasispecies herein analyzed. During virus replication, many generated mutations are not able to generate virus progeny, so they are lost. However, those mutations that are beneficial for viral fitness are naturally selected and will increase their allelic representation in the viral quasispecies (Domingo and Perales, 2019). With this regard, in the present study, most of the substitutions were not detected with an allele frequency greater than 5%, so they do not pose an adaptive evolutionary advantage for the virus. For instance, in the H1N1 subtype in vaccinated animals, only 63% of all nonsynonymous SNVs found did not exceed the 5% of allele frequency. By contrast, 41 (H1N1) and 4 (H3N2) nonsynonymous substitutions with an allele frequency greater than 5% were found in viral samples from vaccinated animals. These substitutions were allocated in all swine IAV proteins, except M1 and NS2 ones. Regarding the polymerase segments (PB2, PB1, PA), 25 nonsynonymous substitutions were found in the H1N1 subtype of vaccinated animals. Single substitutions within these proteins have been related to an increase in IAV polymerase activity, increasing its virulence and facilitating host interspecies jump (Subbarao et al., 1993; Song et al., 2015; Yu et al., 2015; Cai et al., 2020). In the H1N1 NS1 protein, two substitutions were found in the effector binding domain, and both were detected in animal No. 9. Substitution T91I is allocated in the domain that recruits the host eukaryotic translation initiation factor 4G1 (eIF4G), favoring viral mRNA translation instead of host mRNA (de la Luna et al., 1995; Aragón et al., 2000). In addition, substitution L146P is allocated in the nuclear export signal; according to a previous study, leucine at this position is required for NS1 cytoplasmatic localization. Although such substitution was detected in the mentioned study, it was only found on day 5 dpi with an allele frequency of 13% (Li et al., 1998). In relation to the NP, 4 nonsynonymous substitutions were found in the H1N1 subtype, while none were identified in the H3N2 one. These results were detected in previous studies carried out in our group, where the evolutionary capacity of H1N1 and H3N2 subtypes was separately evaluated, and 6 nonsynonymous substitutions were detected in the H1N1 subtype and none in the H3N2 one (López-Valiñas et al., 2021; López-Valiñas et al., 2022).

Surface glycoproteins HA and NA proteins are highly immunogenic, constituting the main target to generate neutralizing antibodies by the host after IAV infection or vaccination (Li et al., 2011; Eichelberger and Wan, 2014; Ma, 2020). Thus, both proteins are under high immune pressure when a previously immunized host becomes infected with the virus, which triggers a greater genetic drift (Li et al., 2011; Krammer, 2019). The HA is a viral surface glycoprotein that attaches to sialic acid allocated in the swine host respiratory tract, allowing virus cell entry (Russell, 2021). In the present study, 6 *de novo* substitutions (E111K, N275S, S278P, T408A, V466I, and K467R) with an allele frequency greater than 5% were detected in vaccinated animals. The E111K in the vestigial esterase subdomain is allocated in a previously described epitope region of the protein(Eisenlohr et al., 1988; Yewdell, 2010). Substitutions N275S and S278P were found in the receptor binding domain (RBD) of the protein, where the main antigenic sites of the virus are allocated (Xu et al., 2010; Guo et al., 2018). It has been already described that single substitutions in this domain are related to the virus interspecies switch (Aytay and Schulze, 1991; Murcia et al., 2012; Thompson and Paulson, 2021). Notably, the N275S substitution was reported with a 97.58% of allele frequency in the vaccinated animal No. 9 at 4 dpi, although its allele frequency decreased to 5.58% in BALF at necropsy of this pig. Finally, the remaining 3 *de novo* substitutions, T408A, V466I, and K467R, were detected on the stalk domain of the protein. Two of these substitutions were also reported from animal No. 4, reaching 36.64% of allele frequency for K467R. In a previous similar experiment, substitutions in this domain were also found in the H3N2 subtype in vaccinated animals, finding exactly one substitution at position 467 (T467I) (López-Valiñas et al., 2022). Notably, relevant nonsynonymous substitutions in vaccinated animals were not found on the H3N2 HA protein.

NA is a surface glycoprotein that plays an important role in virion progeny realizing (McAuley et al., 2019). In vaccinated animals, only two nonsynonymous substitutions, whose allele frequency exceeded 5%, were reported, both in the head domain of the protein. These substitutions, S333G in NA of the H3N2 subtype and V394I in NA of the H1N1 one, were located on the exposed part of the protein. The first one is allocated in the epitopic region F’329–339 (Ge et al., 2022), while V394I is nearby to previously described epitopes(Wan et al., 2015; Jiang et al., 2016). Interestingly, V394I was also detected with 73.7% allelic frequency in viral samples from animal No. 9. Therefore, in this animal, 4 important substitutions have been detected in both surface glycoproteins. This pig, together with animal No. 10, had the highest gross pathological score. Therefore, in this animal, apart from the potential VAERD effect, these mutations may be affecting viral fitness, increasing its replicative capacity, and eventually its virulence. Altogether, all substitutions found in both HA and NA, may have an impact on evading the immune system by the virus, although further analyses with reverse genetic technology would be required (Li et al., 2021).

In conclusion, our findings support the wide capacity of evolution and adaptation to changing environments of swine IAV. In the present study, a lower probability of viral reassortment was observed in vaccinated animals, as has been recently reported (Li et al., 2022). However, the vaccine did not avoid the genetic diversification of swine IAV. Herein, nonsynonymous substitutions were found in viral quasispecies collected in vaccinated animals, and the role that they could play in the evasion of the immune system was hypothesized according to previous literature. Finally, the present study underlines the importance of performing surveillance and genomic studies of swine IAV, as well as increasing the vaccination rate in pigs, to reduce the circulation of the virus in the field as much as possible, reducing the reassortment events and minimizing the risk of pandemics in both, humans, and pigs.

## Data availability statement

The datasets presented in this study can be found in online repositories. The names of the repository/repositories and accession number(s) can be found below: https://www.ncbi.nlm.nih.gov/, PRJNA902942.

## Ethics statement

The animal study was reviewed and approved by Animal ethics committee from the Generalitat de Catalunya.

## Author contributions

Conceptualization: JIN. Methodology: ÁL-V, MV, MW, JIN, LG, and JS. HTS libraries preparation: CC. Bioinformatic analysis: ÁL-V. Animal experiment: GC, JS, and JIN. Formal analysis: ÁL-V and JIN. Resources: AD and JIN. Writing—original draft preparation: ÁL-V. Writing—review and editing: ÁL-V, JS, LG, and JIN. Supervision: JIN. project administration: JIN. Funding acquisition: AD and JIN. All authors contributed to the article and approved the submitted version.

## References

[B1] Andrews S. (2010) FastQC: A quality control tool for high throughput sequence data. Available at: https://www.bioinformatics.babraham.ac.uk/projects/fastqc/ (Accessed September 1, 2021).

[B2] Aragón T. de la Luna S. Novoa I. Carrasco L. Ortín J. Nieto A. (2000). Eukaryotic translation initiation factor 4GI is a cellular target for NS1 protein, a translational activator of influenza virus. Mol. Cell Biol. 20, 6259–6268. doi: 10.1128/MCB.20.17.6259-6268.2000 10938102 PMC86100

[B3] Aytay S. Schulze I. T. (1991). Single amino acid substitutions in the hemagglutinin can alter the host range and receptor binding properties of H1 strains of influenza a virus. J. Virol. 65, 3022–3028. doi: 10.1128/JVI.65.6.3022-3028.1991 2033664 PMC240956

[B4] Belshe R. B. (2005). The origins of pandemic influenza — lessons from the 1918 virus. N Engl J Med. 353, 2209–2211. doi: 10.1056/NEJMP058281 16306515

[B5] Bolger A. M. Lohse M. Usadel B. (2014). Trimmomatic: a flexible trimmer for illumina sequence data. Bioinformatics 30, 2114–2120. doi: 10.1093/bioinformatics/btu170 24695404 PMC4103590

[B6] Borges V. Pinheiro M. Pechirra P. Guiomar R. Gomes J. P. (2018). INSaFLU: an automated open web-based bioinformatics suite “from-reads” for influenza whole-genome-sequencing-based surveillance. Genome Med. 10, 46 doi: 10.1186/S13073-018-0555-0 PMC6027769 29954441

[B7] Breen M. Nogales A. Baker S. F. Martínez-Sobrido L. (2016). Replication-competent influenza a viruses expressing reporter genes. Viruses 179 (8), 179. doi: 10.3390/V8070179 PMC4974514 27347991

[B8] Brown I. H. (2000). The epidemiology and evolution of influenza viruses in pigs. Vet. Microbiol. 74, 29–46. doi: 10.1016/S0378-1135(00)00164-4 10799776

[B9] Brown I. H. Harris P. A. McCauley J. W. Alexander D. J. (1998). Multiple genetic reassortment of avian and human influenza a viruses in European pigs, resulting in the emergence of an H1N2 virus of novel genotype. J. Gen. Virol. 79 (Pt 12), 2947–2955. doi: 10.1099/0022-1317-79-12-2947 9880008

[B10] Busquets N. Segalés J. Córdoba L. Mussá T. Crisci E. Martín-Valls G. E.. (2010). Experimental infection with H1N1 European swine influenza virus protects pigs from an infection with the 2009 pandemic H1N1 human influenza virus. Vet. Res. 41, 74. doi: 10.1051/vetres/2010046 20663475 PMC2939699

[B11] Cacciabue M. Currá A. Carrillo E. König G. Gismondi M. I. (2020). A beginner’s guide for FMDV quasispecies analysis: sub-consensus variant detection and haplotype reconstruction using next-generation sequencing. Brief Bioinform. 21, 1766–1775. doi: 10.1093/bib/bbz086 31697321 PMC7110011

[B12] Cai M. Zhong R. Qin C. Yu Z. Wen X. Xian J.. (2020). The R251K substitution in viral protein PB2 increases viral replication and pathogenicity of Eurasian avian-like H1N1 swine influenza viruses. Viruses 52 (12), 52. doi: 10.3390/V12010052 PMC7019279 31906472

[B13] Carrat F. Flahault A. (2007). Influenza vaccine: The challenge of antigenic drift. Vaccine 25, 6852–6862. doi: 10.1016/j.vaccine.2007.07.027 17719149

[B14] Chamba Pardo F. O. Schelkopf A. Allerson M. Morrison R. Culhane M. Perez A.. (2018). Breed-to-wean farm factors associated with influenza a virus infection in piglets at weaning. Prev. Vet. Med. 161, 33–40. doi: 10.1016/J.PREVETMED.2018.10.008 30466656

[B15] Chen R. Holmes E. C. (2006). Avian influenza virus exhibits rapid evolutionary dynamics. Mol. Biol. Evol. 23, 2336–2341. doi: 10.1093/MOLBEV/MSL102 16945980

[B16] Cingolani P. Platts A. Wang L. L. Coon M. Nguyen T. Wang L.. (2012). A program for annotating and predicting the effects of single nucleotide polymorphisms, SnpEff: SNPs in the genome of drosophila melanogaster strain w1118; iso-2; iso-3. Fly (Austin) 6, 80–92. doi: 10.4161/fly.19695 22728672 PMC3679285

[B17] Danecek P. Bonfield J. K. Liddle J. Marshall J. Ohan V. Pollard M. O.. (2021). Twelve years of SAMtools and BCFtools. Gigascience 10, 1–4. doi: 10.1093/gigascience/giab008 PMC7931819 33590861

[B18] de la Luna S. Fortes P. Beloso A. Ortín J. (1995). Influenza virus NS1 protein enhances the rate of translation initiation of viral mRNAs. J. Virol. 69, 2427–2433. doi: 10.1128/jvi.69.4.2427-2433.1995 7884890 PMC188917

[B19] Detmer S. E. Gunvaldsen R. E. Harding J. C. (2013). Comparison of influenza a virus infection in high- and low-birth-weight pigs using morphometric analysis. Influenza Other Respir. Viruses 7, 2–9. doi: 10.1111/irv.12199 PMC5655882 24224813

[B20] Diaz A. Allerson M. Culhane M. Sreevatsan S. Torremorell M. (2013). Antigenic drift of H1N1 influenza a virus in pigs with and without passive immunity. Influenza Other Respir. Viruses 7 Suppl 4, 52–60. doi: 10.1111/IRV.12190 PMC4942991 24224820

[B21] Diaz A. Enomoto S. Romagosa A. Sreevatsan S. Nelson M. Culhane M.. (2015). Genome plasticity of triple-reassortant H1N1 influenza a virus during infection of vaccinated pigs. J. Gen. Virol. 96, 2982. doi: 10.1099/JGV.0.000258 26251306 PMC4857448

[B22] Díaz I. Gimeno M. Callén A. Pujols J. López S. Charreyre C.. (2013). Comparison of different vaccination schedules for sustaining the immune response against porcine reproductive and respiratory syndrome virus. Vet. J. 197, 438–444. doi: 10.1016/J.TVJL.2013.02.008 23499541

[B23] Domingo E. Perales C. (2019). Viral quasispecies. PloS Genet. 15. doi: 10.1371/JOURNAL.PGEN.1008271 PMC6797082 31622336

[B24] Domingo E. Sheldon J. Perales C. (2012). Viral quasispecies evolution. Microbiol. Mol. Biol. Rev. 76, 159–216. doi: 10.1128/mmbr.05023-11 22688811 PMC3372249

[B25] Eichelberger M. C. Wan H. (2014). Influenza neuraminidase as a vaccine antigen. Curr. Top. Microbiol. Immunol. 386, 275–299. doi: 10.1007/82_2014_398/COVER 25033754

[B26] Eisenlohr L. C. Gerhard W. Hackett C. J. (1988). Acid-induced conformational modification of the hemagglutinin molecule alters interaction of influenza virus with antigen-presenting cells. J. Immunol. (Baltimore, Md. : 1950) 141 (6), 1870–1876. 2459193

[B27] Er C. Lium B. Tavornpanich S. Hofmo P. O. Forberg H. Hauge A. G.. (2014). Adverse effects of influenza A(H1N1)pdm09 virus infection on growth performance of Norwegian pigs - a longitudinal study at a boar testing station. BMC Vet. Res. 10, 284 doi: 10.1186/S12917-014-0284-6 PMC4300606 25472551

[B28] Fitch W. M. Leiter J. M. Li X. Q. Palese P. (1991). Positive Darwinian evolution in human influenza a viruses. Proc. Natl. Acad. Sci. U.S.A. 88, 4270–4274. doi: 10.1073/pnas.88.10.4270 1840695 PMC51640

[B29] Frost S. D. W. Magalis B. R. Kosakovsky Pond S. L. (2018). Neutral theory and rapidly evolving viral pathogens. Mol. Biol. Evol. 35, 1348–1354. doi: 10.1093/MOLBEV/MSY088 29688481 PMC6279309

[B30] Galindo-Cardiel I. Ballester M. Solanes D. Nofrarías M. López-Soria S. Argilaguet J. M.. (2013). Standardization of pathological investigations in the framework of experimental ASFV infections. Virus Res. 173, 180–190. doi: 10.1016/j.virusres.2012.12.018 23313935

[B31] Ganges L. Núñez J. I. Sobrino F. Borrego B. Fernández-Borges N. Frías-Lepoureau M. T.. (2008). Recent advances in the development of recombinant vaccines against classical swine fever virus: cellular responses also play a role in protection. Vet. J. 177, 169–177. doi: 10.1016/J.TVJL.2007.01.030 17804267

[B32] Gauger P. C. Vincent A. L. Loving C. L. Lager K. M. Janke B. H. Kehrli M. E.. (2011). Enhanced pneumonia and disease in pigs vaccinated with an inactivated human-like (δ-cluster) H1N2 vaccine and challenged with pandemic 2009 H1N1 influenza virus. Vaccine 29, 2712–2719. doi: 10.1016/J.VACCINE.2011.01.082 21310191

[B33] Ge J. Lin X. Guo J. Liu L. Li Z. Lan Y.. (2022). The antibody response against neuraminidase in human influenza a (H3N2) virus infections during 2018/2019 flu season: Focusing on the epitopes of 329-N-Glycosylation and E344 in N2. Front. Microbiol. 13. doi: 10.3389/FMICB.2022.845088/BIBTEX PMC8978628 35387078

[B34] Guo Z. Wilson J. R. York I. A. Stevens J. (2018). Biosensor-based epitope mapping of antibodies targeting the hemagglutinin and neuraminidase of influenza a virus. J. Immunol. Methods 461, 23–29. doi: 10.1016/J.JIM.2018.07.007 30053389 PMC6416777

[B35] Hu Y. J. Tu P. C. Lin C. S. Guo S. T. (2014). Identification and chronological analysis of genomic signatures in influenza a viruses. PloS One 9. doi: 10.1371/journal.pone.0084638 PMC3885579 24416256

[B36] Image J. Available at: https://imagej.nih.gov/ij/ (Accessed January 14, 2022).

[B37] Ito T. Couceiro J. N. S. S. Kelm S. Baum L. G. Krauss S. Castrucci M. R.. (1998). Molecular basis for the generation in pigs of influenza a viruses with pandemic potential. J. Virol. 72, 7367–7373. doi: 10.1128/JVI.72.9.7367-7373.1998 9696833 PMC109961

[B38] Jiang L. Fantoni G. Couzens L. Gao J. Plant E. Ye Z.. (2016). Comparative efficacy of monoclonal antibodies that bind to different epitopes of the 2009 pandemic H1N1 influenza virus neuraminidase. J. Virol. 90, 117–128. doi: 10.1128/JVI.01756-15/ASSET/85EF0ACA-5BAA-4F00-A66D-F8D6358AFF8D/ASSETS/GRAPHIC/ZJV9990910630005.JPEG 26468531 PMC4702561

[B39] Kawaoka Y. Krauss S. Webster R. G. (1989). Avian-to-human transmission of the PB1 gene of influenza a viruses in the 1957 and 1968 pandemics. J. Virol. 63, 4608. doi: 10.1128/JVI.63.11.4603-4608.1989 PMC251093 2795713

[B40] Kim M. Cheong Y. Lee J. Lim J. Byun S. Jang Y. H.. (2021). A host-restricted self-attenuated influenza virus provides broad pan-influenza a protection in a mouse model. Front. Immunol. 12. doi: 10.3389/FIMMU.2021.779223 PMC8674563 34925355

[B41] Kimble J. B. Wymore Brand M. Kaplan B. S. Gauger P. Coyle E. M. Chilcote K.. (2022). Vaccine-associated enhanced respiratory disease following influenza virus infection in ferrets recapitulates the model in pigs. J. Virol. 96. doi: 10.1128/JVI.01725-21/ASSET/428F8F3C-3754-4717-AA6E-1ADA592C7ACC/ASSETS/IMAGES/LARGE/JVI.01725-21-F006.JPG PMC8906406 34985999

[B42] Krammer F. (2019). The human antibody response to influenza a virus infection and vaccination. Nat. Rev. Immunol. 19, 6 19, 383–397. doi: 10.1038/s41577-019-0143-6 30837674

[B43] Kyriakis C. S. Rose N. Foni E. Maldonado J. Loeffen W. L. A. Madec F.. (2013). Influenza a virus infection dynamics in swine farms in Belgium, France, Italy and spain 2006-2008. Vet. Microbiol. 162, 543–550. doi: 10.1016/J.VETMIC.2012.11.014 23201246

[B44] Li C. Culhane M. R. Schroeder D. C. Cheeran M. C. J. Galina Pantoja L. Jansen M. L.. (2022). Vaccination decreases the risk of influenza a virus reassortment but not genetic variation in pigs. Elife 11. doi: 10.7554/ELIFE.78618 PMC9439680 36052992

[B45] Li H. Durbin R. (2009). Fast and accurate short read alignment with burrows-wheeler transform. Bioinformatics 25, 1754–1760. doi: 10.1093/BIOINFORMATICS/BTP324 19451168 PMC2705234

[B46] Li Y. Robertson I. (2021). The epidemiology of swine influenza. Anim. Dis., 1(1),1–14. doi: 10.1186/S44149-021-00024-6 PMC8476212 34778883

[B47] Li W. Shi W. Qiao H. Ho S. Y. W. Luo A. Zhang Y.. (2011). Positive selection on hemagglutinin and neuraminidase genes of H1N1 influenza viruses. Virol. J. 8, 183 doi: 10.1186/1743-422X-8-183 PMC3094300 21507270

[B48] Li Y. Yamakita Y. Krug R. M. (1998). Regulation of a nuclear export signal by an adjacent inhibitory sequence: The effector domain of the influenza virus NS1 protein. Proc. Natl. Acad. Sci. 95, 4864–4869. doi: 10.1073/PNAS.95.9.4864 9560194 PMC20179

[B49] Li Z. Zhong L. He J. Huang Y. Zhao Y. (2021). Development and application of reverse genetic technology for the influenza virus. Virus Genes 57, 151. doi: 10.1007/S11262-020-01822-9 33528730 PMC7851324

[B50] López-Serrano S. Cordoba L. Pérez-Maillo M. Pleguezuelos P. Remarque E. J. Ebensen T.. (2021). Immune responses to pandemic H1N1 influenza virus infection in pigs vaccinated with a conserved hemagglutinin HA1 peptide adjuvanted with CAF®01 or CDA/αGalCerMPEG. Vaccines (Basel) 9, 751. doi: 10.3390/vaccines9070751 34358167 PMC8310093

[B51] López-Valiñas Á. Baioni L. Córdoba L. Darji A. Chiapponi C. Segalés J.. (2022). Evolution of swine influenza virus H3N2 in vaccinated and nonvaccinated pigs after previous natural H1N1 infection. Viruses 14. doi: 10.3390/V14092008/S1 PMC9505157 36146814

[B52] López-Valiñas Á. Sisteré-Oró M. López-Serrano S. Baioni L. Darji A. Chiapponi C.. (2021). Identification and characterization of swine influenza virus h1n1 variants generated in vaccinated and nonvaccinated, challenged pigs. Viruses 13, 2087. doi: 10.3390/V13102087/S1 34696517 PMC8539973

[B53] Lycett S. J. Baillie G. Coulter E. Bhatt S. Kellam P. McCauley J. W.. (2012). Estimating reassortment rates in co-circulating Eurasian swine influenza viruses. J. Gen. Virol. 93, 2326–2336. doi: 10.1099/vir.0.044503-0 22971819 PMC3542128

[B54] Ma W. (2020). Swine influenza virus: Current status and challenge (Elsevier B.V). doi: 10.1016/j.virusres.2020.198118 PMC7587018 32798539

[B55] Ma W. Kahn R. E. Richt J. A. (2009). The pig as a mixing vessel for influenza viruses: Human and veterinary implications. J. Mol. Genet. Med. 3, 158. doi: 10.4172/1747-0862.1000028 PMC2702078 19565018

[B56] Ma W. Richt J. A. (2010). Swine influenza virus: Current status and challenge. Virus Res. 288, 198118. doi: 10.1017/S146625231000006X PMC7587018 32798539

[B57] Martín-Valls G. E. Simon-Grifé M. van Boheemen S. de Graaf M. Bestebroer T. M. Busquets N.. (2014). Phylogeny of Spanish swine influenza viruses isolated from respiratory disease outbreaks and evolution of swine influenza virus within an endemically infected farm. Vet. Microbiol. 170, 266–277. doi: 10.1016/j.vetmic.2014.02.031 24685238

[B58] McAuley J. L. Gilbertson B. P. Trifkovic S. Brown L. E. McKimm-Breschkin J. L. (2019). Influenza virus neuraminidase structure and functions. Front. Microbiol. 0. doi: 10.3389/FMICB.2019.00039 PMC6362415 30761095

[B59] Medina R. A. (2018). 1918 influenza virus: 100 years on, are we prepared against the next influenza pandemic? Nat. Rev. Microbiol. 16, 2 16, 61–62. doi: 10.1038/nrmicro.2017.174 29332942 PMC8628252

[B60] Murcia P. R. Hughes J. Battista P. Lloyd L. Baillie G. J. Ramirez-Gonzalez R. H.. (2012). Evolution of an Eurasian avian-like influenza virus in naïve and vaccinated pigs. PloS Pathog. 8, e1002730. doi: 10.1371/journal.ppat.1002730 22693449 PMC3364949

[B61] Mussá T. Ballester M. Silva-Campa E. Baratelli M. Busquets N. Lecours M. P.. (2013). Swine, human or avian influenza viruses differentially activates porcine dendritic cells cytokine profile. Vet. Immunol. Immunopathol. 154, 25–35. doi: 10.1016/J.VETIMM.2013.04.004 23689011

[B62] Nelson C. W. Moncla L. H. Hughes A. L. (2015). SNPGenie: Estimating evolutionary parameters to detect natural selection using pooled next-generation sequencing data. Bioinformatics 31, 3709–3711. doi: 10.1093/bioinformatics/btv449 26227143 PMC4757956

[B63] Ou J. Zhu L. J. (2019). trackViewer: a bioconductor package for interactive and integrative visualization of multi-omics data. Nat. Methods 16, 6 16, 453–454. doi: 10.1038/s41592-019-0430-y 31133757

[B64] Park J. K. Xiao Y. Ramuta M. D. Rosas L. A. Fong S. Matthews A. M.. (2020). Pre-existing immunity to influenza virus hemagglutinin stalk might drive selection for antibody-escape mutant viruses in a human challenge model. Nat. Med. 26, 8 26, 1240–1246. doi: 10.1038/s41591-020-0937-x PMC7450362 32601336

[B65] Pliasas V. C. Menne Z. Aida V. Yin J. H. Naskou M. C. Neasham P. J.. (2022). A novel neuraminidase virus-like particle vaccine offers protection against heterologous H3N2 influenza virus infection in the porcine model. Front. Immunol. 13. doi: 10.3389/FIMMU.2022.915364 PMC9300842 35874791

[B66] Reed L. J. Muench H. (1938). A simple method of estimating fifty per cent endpoints. Antioch Rev. 27, 493–497. doi: 10.7723/antiochreview.72.3.0546

[B67] Reeth K. Vincent A. L. Lager K. M. (2016). “Vaccines and vaccination for swine influenza: differing situations in Europe and the USA,” in Animal influenza (Hoboken, NJ, USA: John Wiley & Sons, Inc), 480–501. doi: 10.1002/9781118924341.ch19

[B68] RStudio | Open source & professional software for data science teams - RStudio. Available at: https://www.rstudio.com/ (Accessed September 1, 2021).

[B69] Russell C. J. (2021). Hemagglutinin stability and its impact on influenza a virus infectivity, pathogenicity, and transmissibility in avians, mice, swine, seals, ferrets, and humans. Viruses 13 (5), 746. doi: 10.3390/V13050746 PMC8145662 33923198

[B70] Sabattini E. Bisgaard K. Ascani S. Poggi S. Piccioli M. Ceccarelli C.. (1998). The EnVision(TM)+ system: A new immunohistochemical method for diagnostics and research. critical comparison with the APAAP, ChemMate(TM), CSA, LABC, and SABC techniques. J. Clin. Pathol. 51, 506–511. doi: 10.1136/jcp.51.7.506 9797726 PMC500802

[B71] Salvesen H. A. Whitelaw C. B. A. (2021). Current and prospective control strategies of influenza a virus in swine. Porcine Health Manag 7, 23 doi: 10.1186/S40813-021-00196-0 PMC7917534 33648602

[B72] Scholtissek C. Rohde W. von Hoyningen V. Rott R. (1978). On the origin of the human influenza virus subtypes H2N2 and H3N2. Virology 87, 13–20. doi: 10.1016/0042-6822(78)90153-8 664248

[B73] Shao W. Li X. Goraya M. U. Wang S. Chen J. L. (2017). Evolution of influenza a virus by mutation and re-assortment. Int. J. Mol. Sci. 18. doi: 10.3390/ijms18081650 PMC5578040 28783091

[B74] Sibila M. Aragón V. Fraile L. Segalés J. (2014). Comparison of four lung scoring systems for the assessment of the pathological outcomes derived from actinobacillus pleuropneumoniae experimental infections. BMC Vet. Res. 10,165 doi: 10.1186/1746-6148-10-165 PMC4112831 25038822

[B75] Simon G. Larsen L. E. Dürrwald R. Foni E. Harder T. van Reeth K.. (2014). European Surveillance network for influenza in pigs: surveillance programs, diagnostic tools and swine influenza virus subtypes identified in 14 European countries from 2010 to 2013. PloS One 9, e115815. doi: 10.1371/journal.pone.0115815 25542013 PMC4277368

[B76] Simon-Grifé M. Martín-Valls G. E. Vilar M. J. García-Bocanegra I. Mora M. Martín M.. (2011). Seroprevalence and risk factors of swine influenza in Spain. Vet. Microbiol. 149, 56–63. doi: 10.1016/J.VETMIC.2010.10.015 21112702

[B77] Sisteré-Oró M. López-Serrano S. Veljkovic V. Pina-Pedrero S. Vergara-Alert J. Córdoba L.. (2019). DNA Vaccine based on conserved HA-peptides induces strong immune response and rapidly clears influenza virus infection from vaccinated pigs. PloS One 14. doi: 10.1371/journal.pone.0222201 PMC6760788 31553755

[B78] Sitaras I. Kalthoff D. Beer M. Peeters B. de Jong M. C. M. (2014). Immune escape mutants of highly pathogenic avian influenza H5N1 selected using polyclonal sera: Identification of key amino acids in the HA protein. PloS One 9, e84628. doi: 10.1371/JOURNAL.PONE.0084628 24586231 PMC3934824

[B79] Sitaras I. Spackman E. de Jong M. C. M. Parris D. J. (2020). Selection and antigenic characterization of immune-escape mutants of H7N2 low pathogenic avian influenza virus using homologous polyclonal sera. Virus Res. 290, 198188. doi: 10.1016/J.VIRUSRES.2020.198188 33045306

[B80] Smith G. J. D. Vijaykrishna D. Bahl J. Lycett S. J. Worobey M. Pybus O. G.. (2009). Origins and evolutionary genomics of the 2009 swine-origin H1N1 influenza a epidemic. Nature 459, 1122–1125. doi: 10.1038/nature08182 19516283

[B81] Sobel Leonard A. McClain M. T. Smith G. J. D. Wentworth D. E. Halpin R. A. Lin X.. (2016). Deep sequencing of influenza a virus from a human challenge study reveals a selective bottleneck and only limited intrahost genetic diversification. J. Virol. 90, 11247–11258. doi: 10.1128/jvi.01657-16 27707932 PMC5126380

[B82] Song J. Xu J. Shi J. Li Y. Chen H. (2015). Synergistic effect of S224P and N383D substitutions in the PA of H5N1 avian influenza virus contributes to mammalian adaptation. Sci. Rep., 1(5), 1–7. doi: 10.1038/srep10510 PMC4441148 26000865

[B83] Spackman E. Senne D. A. Myers T. J. Bulaga L. L. Garber L. P. Perdue M. L.. (2002). Development of a real-time reverse transcriptase PCR assay for type a influenza virus and the avian H5 and H7 hemagglutinin subtypes. J. Clin. Microbiol. 40, 3256–3260. doi: 10.1128/JCM.40.9.3256-3260.2002 12202562 PMC130722

[B84] Sriwilaijaroen N. Suzuki Y. (2012). Molecular basis of the structure and function of H1 hemagglutinin of influenza virus. Proc. Jpn Acad. Ser. B Phys. Biol. Sci. 88, 226–249. doi: 10.2183/pjab.88.226 PMC3410141 22728439

[B85] Subbarao E. K. London W. Murphy B. R. (1993). A single amino acid in the PB2 gene of influenza a virus is a determinant of host range. J. Virol. 67, 1761–1764. doi: 10.1128/JVI.67.4.1761-1764.1993 8445709 PMC240216

[B86] Sun H. Xiao Y. Liu J. Wang D. Li F. Wang C.. (2020). Prevalent Eurasian avian-like H1N1 swine influenza virus with 2009 pandemic viral genes facilitating human infection. Proc. Natl. Acad. Sci. U.S.A. 117, 17204–17210. doi: 10.1073/pnas.1921186117 32601207 PMC7382246

[B87] Swine influenza - WOAH - World Organisation for Animal Health. Available at: https://www.woah.org/en/disease/swine-influenza/ (Accessed August 4, 2022).

[B88] Tarradas J. de la Torre M. E. Rosell R. Perez L. J. Pujols J. Muñoz M.. (2014). The impact of CSFV on the immune response to control infection. Virus Res. 185, 82–91. doi: 10.1016/J.VIRUSRES.2014.03.004 24657786

[B89] Thacker E. Janke B. (2008). Swine influenza virus: Zoonotic potential and vaccination strategies for the control of avian and swine influenzas. J. Infect. Dis. 197, S19–S24. doi: 10.1086/524988 18269323

[B90] Thompson A. J. Paulson J. C. (2021). Adaptation of influenza viruses to human airway receptors. J. Biol. Chem. 296, 100017. doi: 10.1074/JBC.REV120.013309 33144323 PMC7948470

[B91] Torremorell M. Allerson M. Corzo C. Diaz A. Gramer M. (2012). Transmission of influenza a virus in pigs. Transbound Emerg. Dis. 59, 68–84. doi: 10.1111/J.1865-1682.2011.01300.X 22226050

[B92] Trifonov V. Khiabanian H. Rabadan R. (2009). Geographic dependence, surveillance, and origins of the 2009 influenza a (H1N1) virus. N Engl J Med. 361, 115–119. doi: 10.1056/NEJMP0904572 19474418

[B93] van der Vries E. Collins P. J. Vachieri S. G. Xiong X. Liu J. Walker P. A.. (2012). H1N1 2009 pandemic influenza virus: Resistance of the I223R neuraminidase mutant explained by kinetic and structural analysis. PloS Pathog. 8. doi: 10.1371/journal.ppat.1002914 PMC3447749 23028314

[B94] Vincent A. L. Lager K. M. Janke B. H. Gramer M. R. Richt J. A. (2008). Failure of protection and enhanced pneumonia with a US H1N2 swine influenza virus in pigs vaccinated with an inactivated classical swine H1N1 vaccine. Vet. Microbiol. 126, 310–323. doi: 10.1016/J.VETMIC.2007.07.011 17719188

[B95] Wan H. Yang H. Shore D. A. Garten R. J. Couzens L. Gao J.. (2015). Structural characterization of a protective epitope spanning A(H1N1)pdm09 influenza virus neuraminidase monomers. Nat. Commun. 6, 1 6, 1–1 6,10. doi: 10.1038/ncomms7114 PMC4347215 25668439

[B96] Wickham H. (2016). Ggplot2: Elegant graphics for data analysis (New York: Springer-Verlag). doi: 10.1007/978-0-387-98141-3

[B97] Wilm A. Aw P. P. K. Bertrand D. Yeo G. H. T. Ong S. H. Wong C. H.. (2012). LoFreq: a sequence-quality aware, ultra-sensitive variant caller for uncovering cell-population heterogeneity from high-throughput sequencing datasets. Nucleic Acids Res. 40, 11189. doi: 10.1093/NAR/GKS918 23066108 PMC3526318

[B98] Xu R. Ekiert D. C. Krause J. C. Hai R. Crowe J. E. Wilson I. A. (2010). Structural basis of preexisting immunity to the 2009 H1N1 pandemic influenza virus. Science 328, 357–360. doi: 10.1126/science.1186430 20339031 PMC2897825

[B99] Xu C. Zhang N. Yang Y. Liang W. Zhang Y. Wang J.. (2022). Immune escape adaptive mutations in hemagglutinin are responsible for the antigenic drift of Eurasian avian-like H1N1 swine influenza viruses. J. Virol. 96. doi: 10.1128/JVI.00971-22/ASSET/CFE88D01-46C6-4318-805E-8D6BDCE51EB5/ASSETS/IMAGES/LARGE/JVI.00971-22-F004.JPG PMC9400474 35916512

[B100] Yasuhara A. Yamayoshi S. Soni P. Takenaga T. Kawakami C. Takashita E.. (2017). Diversity of antigenic mutants of influenza A(H1N1)pdm09 virus escaped from human monoclonal antibodies. Sci. Rep. 7, 1 7, 1–1 7, 9. doi: 10.1038/s41598-017-17986-8 29255273 PMC5735164

[B101] Yewdell J. W. (2010). Monoclonal antibodies specific for discontinuous epitopes direct refolding of influenza a virus hemagglutinin. Mol. Immunol. 47, 1132–1136. doi: 10.1016/J.MOLIMM.2009.10.023 20047763 PMC2814887

[B102] Yu Z. Cheng K. Sun W. Zhang X. Li Y. Wang T.. (2015). A PB1 T296R substitution enhance polymerase activity and confer a virulent phenotype to a 2009 pandemic H1N1 influenza virus in mice. Virology 486, 180–186. doi: 10.1016/J.VIROL.2015.09.014 26453960

[B103] Zharikova D. Mozdzanowska K. Feng J. Zhang M. Gerhard W. (2005). Influenza type a virus escape mutants emerge *In vivo* in the presence of antibodies to the ectodomain of matrix protein 2. J. Virol. 79, 6644–6654. doi: 10.1128/JVI.79.11.6644-6654.2005/ASSET/88AD9789-EC47-4B46-86DA-635D0D9B6BA8/ASSETS/GRAPHIC/ZJV0110563030008.JPEG 15890902 PMC1112148

[B104] Zhou B. Donnelly M. E. Scholes D. T. st. George K. Hatta M. Kawaoka Y.. (2009). Single-reaction genomic amplification accelerates sequencing and vaccine production for classical and swine origin human influenza a viruses. J. Virol. 83, 10309–10313. doi: 10.1128/jvi.01109-09 19605485 PMC2748056

